# The neuronal receptor tyrosine kinase Alk is a target for longevity

**DOI:** 10.1111/acel.13137

**Published:** 2020-04-15

**Authors:** Nathaniel S. Woodling, Benjamin Aleyakpo, Miranda Claire Dyson, Lucy J. Minkley, Arjunan Rajasingam, Adam J. Dobson, Kristie H. C. Leung, Simona Pomposova, Matías Fuentealba, Nazif Alic, Linda Partridge

**Affiliations:** ^1^ Department of Genetics, Evolution and Environment Institute of Healthy Ageing University College London London UK; ^2^ Max Planck Institute for Biology of Ageing Cologne Germany; ^3^ Present address: College of Medical, Veterinary and Life Sciences Institute of Molecular, Cell and Systems Biology University of Glasgow Davidson Building Glasgow G12 8QQ UK

**Keywords:** aging, Alk, *Drosophila*, insulin/IGF signalling, lifespan, nervous system, receptor tyrosine kinase

## Abstract

Inhibition of signalling through several receptor tyrosine kinases (RTKs), including the insulin‐like growth factor receptor and its orthologues, extends healthy lifespan in organisms from diverse evolutionary taxa. This raises the possibility that other RTKs, including those already well studied for their roles in cancer and developmental biology, could be promising targets for extending healthy lifespan. Here, we focus on anaplastic lymphoma kinase (Alk), an RTK with established roles in nervous system development and in multiple cancers, but whose effects on aging remain unclear. We find that several means of reducing Alk signalling, including mutation of its ligand jelly belly (jeb), RNAi knock‐down of Alk, or expression of dominant‐negative Alk in adult neurons, can extend healthy lifespan in female, but not male, *Drosophila*. Moreover, reduced Alk signalling preserves neuromuscular function with age, promotes resistance to starvation and xenobiotic stress, and improves night sleep consolidation. We find further that inhibition of Alk signalling in adult neurons modulates the expression of several insulin‐like peptides, providing a potential mechanistic link between neuronal Alk signalling and organism‐wide insulin‐like signalling. Finally, we show that TAE‐684, a small molecule inhibitor of Alk, can extend healthy lifespan in *Drosophila*, suggesting that the repurposing of Alk inhibitors may be a promising direction for strategies to promote healthy aging.

## Graphical abstract

The receptor tyrosine kinase Alk is expressed primarily in neurons, where its signalling overlaps with that of the lifespan‐modulating insulin signalling pathway. Here, we find that Alk inhibition extends lifespan, preserves locomotor function and improves stress resistance in female fruit flies, potentially via a mechanism involving insulin‐like peptide signalling from neurons to and from adipose tissues. These findings pave the way for future studies on Alk and aging in more complex animals.

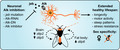

## 1 INTRODUCTION

Human life expectancy in developed countries has risen steadily at the astonishing rate of 3 months per year over the past 160 years (Oeppen & Vaupel, 2002), with a similar rate of increase predicted until at least 2030 (Kontis et al., 2017). This rapid expansion of an older population has led to an increased societal burden of care, underscoring the need to understand biological mechanisms of healthy aging that can be harnessed to improve health in older ages. To this end, the past few decades of research have uncovered several key conserved hallmarks that both characterize aging and have the capacity to modulate healthy lifespan (López‐Otín, Blasco, Partridge, Serrano, & Kroemer, 2013). Among these hallmarks, disrupted nutrient sensing pathways and intercellular communication have been highlighted by findings that several conserved signalling proteins in nutrient sensing pathways can modulate healthy lifespan. Many of these proteins lie in pathways downstream of receptor tyrosine kinases (RTKs) that coordinate not only growth and development but also adult metabolism and function.

For example, the insulin‐like growth factor (IGF) receptor is an RTK whose signalling pathways have been extensively studied for their capacity to modulate healthy lifespan among diverse eukaryotic species (Alic & Partridge, 2011; Fontana, Partridge, & Longo, 2010). Notably, multiple interventions that inhibit insulin/IGF signalling (IIS) can extend healthy lifespan. At the level of the receptors, extension of healthy lifespan has been observed for reduced production of insulin‐like ligands in the vinegar fly *Drosophila* (Broughton et al., 2005; Grönke, Clarke, Broughton, Andrews, & Partridge, 2010), reduced signalling through the insulin/IGF receptor orthologue or its substrates in *Drosophila* (Clancy et al., 2001; Slack et al., 2010; Tatar et al., 2001), heterozygous deletion of the IGF‐1 receptor in mice (Holzenberger et al., 2003), and homozygous deletion of the insulin receptor substrate Irs1 in mice (Selman et al., 2008). Downstream of RTKs, lifespan extension has been reported in *Drosophila* with inhibited function of the effector kinases PI3K or Ras (Slack et al., 2015; Slack, Giannakou, Foley, Goss, & Partridge, 2011), or over‐expression of the transcription factor Foxo, whose activity is inhibited by IIS (Giannakou et al., 2004; Hwangbo et al., 2004). Excitingly, these pathways appear important for human longevity as well: candidate gene studies in centenarians have found enrichment for single‐nucleotide polymorphisms in genes encoding the IGF‐1 receptor (Suh et al., 2008) and Foxo3a (Flachsbart et al., 2009; Willcox et al., 2008). These studies suggest that RTK‐mediated signalling pathways are a promising direction for understanding aging across species and for uncovering therapeutic targets that can modulate the aging process itself.

While IIS has been a critical gateway for understanding the modulation of healthy aging, the possibility remains that other RTKs can exert similar effects. In humans, 58 RTKs have been identified with distinct ligands, tissue expression patterns and physiological functions (Lemmon & Schlessinger, 2010). In *Drosophila*, at least 20 genes encoding RTKs exist in the genome, most with clear mammalian orthologues; however, even in the well‐studied field of *Drosophila* development, the function of many of these RTKs remains unclear (Sopko & Perrimon, 2013), and few have been studied for their roles in aging. In the field of cancer biology, however, a recurring role for mutations in many RTKs has made them a focus for a great deal of translational research. Among these, mutations in anaplastic lymphoma kinase (Alk) have been associated with lymphoma, neuroblastoma and non‐small‐cell lung cancers (Hallberg & Palmer, 2013), leading to the development of effective small molecule Alk inhibitors for clinical use (Kwak et al., 2010; Peters et al., 2017). This critical role of Alk in tumorigenesis has spurred a growing number of studies aiming to understand not only its pathological potentials but also its physiological functions.

Under basal conditions, Alk is expressed most highly in the nervous system, both in vertebrates, including zebrafish (Yao et al., 2013), and in invertebrates, including *Drosophila* (Cheng et al., 2011). In vertebrates, recent studies have identified two activating ligands, ALKAL1 and ALKAL2 (Fadeev et al., 2018; Guan et al., 2015), whereas in *Drosophila* the single identified ligand is the secreted LDL repeat protein jelly belly (jeb) (Englund et al., 2003). Alk signalling is essential for a number of developmental processes: proper neuronal differentiation and survival in zebrafish (Yao et al., 2013), sparing of nervous system growth during nutrient deprivation in larval *Drosophila* (Cheng et al., 2011), regulation of body growth during nutrient deprivation in larval *Drosophila* (Okamoto & Nishimura, 2015), and neuronal circuit assembly in the developing *Drosophila* retina (Bazigou et al., 2007) and neuromuscular junction (Rohrbough & Broadie, 2010).

Alk signalling also plays important roles in adult nervous system function. Adult‐onset Alk inhibition in neurons enhances associative memory in both wild‐type and neurofibromatosis type 1 (NF1) disease model *Drosophila* (Gouzi, Bouraimi, Roussou, Moressis, & Skoulakis, 2018; Gouzi et al., 2011), and Alk knockout in mice increases adult hippocampal neurogenesis and enhances performance in novel object recognition tasks (Bilsland et al., 2008). These findings have led to the hypothesis that, in addition to its more canonical roles as an RTK in growth and nutrient sensing, Alk plays a specific role in constraining long‐term memory formation (Gouzi et al., 2018). These findings raise the possibility that other functions remain to be identified for Alk in the adult brain.

Here, we have asked whether Alk, like several other RTKs, modulates healthy lifespan in *Drosophila*. We build on several established tools to inhibit Alk signalling, including mutation of the *jeb* gene, RNAi knock‐down of Alk, and expression of a dominant‐negative Alk protein in adult neurons. In each case, we find that Alk inhibition can extend healthy lifespan in flies. Moreover, we find that inhibition of Alk signalling improves neuromuscular function of aging flies, extends survival under starvation or xenobiotic stressors, and improves night sleep consolidation. Finally, we report that TAE‐684, a small molecule Alk inhibitor, can extend healthy lifespan in *Drosophila*, suggesting that Alk may be a promising pharmacological target in aging.

## 2 RESULTS

### 2.1 Reduced jeb expression extends lifespan

**Figure 1:**
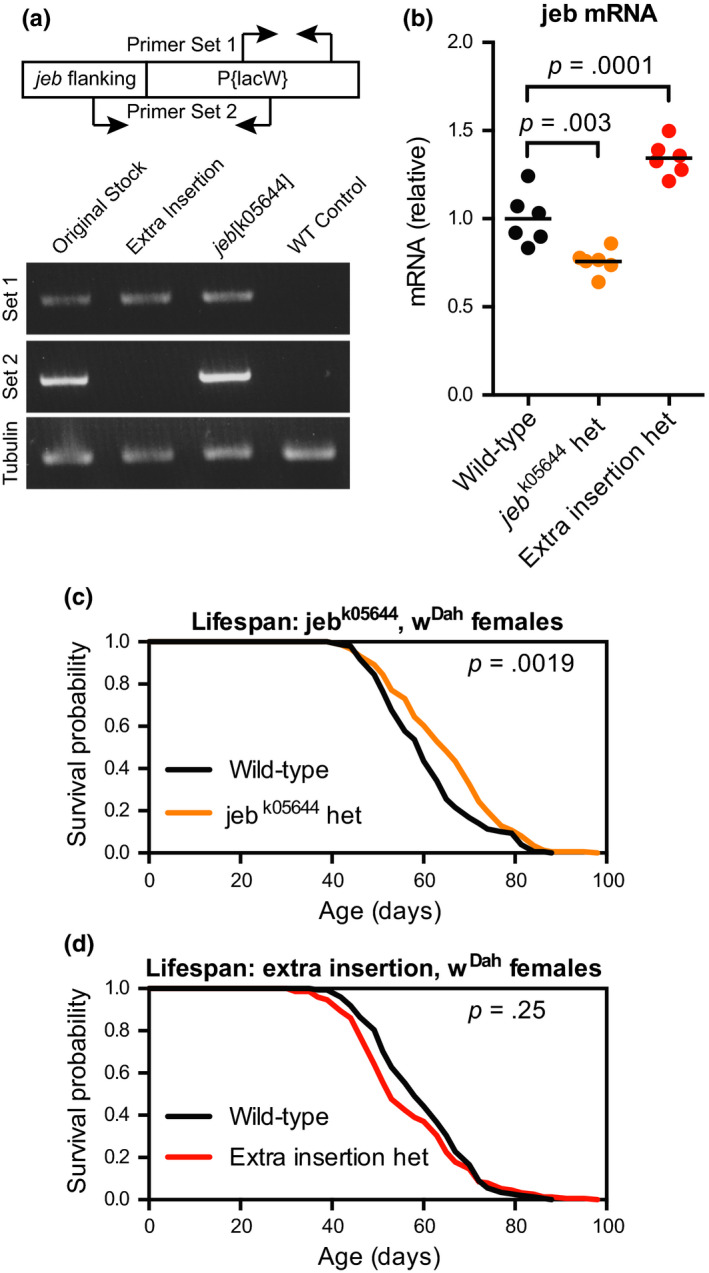
Heterozygous loss of *jeb* extends lifespan. (a) The original stock (BL10576) containing the jeb^k05644^ mutation contained two insertions of P{lacW} that became apparent upon back‐crossing. Primers designed as shown amplified either P{lacW} independent of its insertion site (Set 1) or P{lacW} in the annotated insertion site in the *jeb* gene (Set 2). PCR from genomic DNA using these primers confirmed the presence of the jeb^k05644^ insertion or the secondary extra insertion in each back‐crossed stock. (b) qPCR from whole‐fly RNA showed reduced *jeb* mRNA levels in female w^Dah^;jeb^k05644^/+; flies compared to w^Dah^;+/+; flies, with increased *jeb* mRNA levels in w^Dah^;extra‐insertion/+; flies compared to w^Dah^;+/+; flies. *n* = 6 biological replicates of three whole flies per replicate for each genotype; *p* values are from Dunnett's multiple comparison tests between the indicated groups. (c) Survival curves showed extended lifespan for female w^Dah^;jeb^k05644^/+; flies compared to their w^Dah^;+/+; siblings. (d) Survival curves showed no significant change in lifespan for female w^Dah^;extra‐insertion/+; flies compared to their w^Dah^;+/+; siblings. For all survival experiments, *n* > 145 deaths counted per condition; *p* values are from log‐rank tests vs. the wild‐type condition

The *jelly belly* (*jeb*) gene encodes the single activating ligand for Alk in *Drosophila* (Englund et al., 2003). Because deletion of other RTK ligands in Drosophila has previously been shown to extend healthy lifespan (Grönke et al., 2010), we asked whether deletion of *jeb* would have a similar effect on lifespan. The *jeb^k05644^* allele has been well characterized as a *jeb* loss‐of‐function allele due to insertion of the P{lacW} element in the first intron of *jeb* (Weiss, Suyama, Lee, & Scott, 2001). Flies carrying this allele are healthy and viable as heterozygotes, but homozygotes show early larval lethality due to failed fusion of the visceral mesoderm (Englund et al., 2003; Weiss et al., 2001). We obtained the corresponding Bloomington Stock Center strain (BL10576), and upon back‐crossing the strain to standardize genetic background, we observed that the stock contained two insertions of P{lacW} on the second chromosome, one whose *mini‐white* transgene produced an orange eye colour and another whose *mini‐white* transgene produced a red eye colour. We isolated each insertion as an independent strain, and both were homozygous lethal. We determined by PCR from genomic DNA that only the orange‐eye‐producing insertion was within the expected location in the *jeb* gene (Figure 1a). We confirmed by quantitative real‐time PCR (qPCR) that the isolated *jeb^k05644^* allele produced a significant ~25% reduction in *jeb* mRNA levels in heterozygotes (Figure 1b). Notably, the independent insertion of P{lacW} produced a significant ~35% increase in *jeb* mRNA levels, potentially due to selection for genetic modifiers that could compensate for partial loss of *jeb* in the original stock.

We next tested whether heterozygous loss of *jeb* would extend healthy lifespan. We found that heterozygous *jeb^k05644^* female flies were significantly long‐lived compared to their wild‐type siblings (Figure 1c, median lifespan + 8.3% and *p* = .0019 vs. wild‐type). This lifespan extension was reproducible in independent experiments in female flies but not in males (Figure S1). This sexual dimorphism in lifespan extension is reminiscent of other studies reporting greater lifespan extensions in females than in males for rapamycin treatment in flies (Bjedov et al., 2010), dietary restriction in flies (Regan et al., 2016), S6K1 mutation in mice (Selman et al., 2009), and lowered insulin‐like signalling in both flies (Clancy et al., 2001; Tatar et al., 2001) and mice (Selman et al., 2008). Finally, as a control, we found no significant change in lifespan in female flies heterozygous for the extra insertion of P{lacW} compared to their wild‐type siblings (Figure 1d, median lifespan −8.6% and *p* = .25 vs. wild‐type). While not significant, these flies were, if anything short‐lived, consistent with the increased expression of *jeb*. These results suggest that reduced levels of jeb, and therefore reduced signalling through Alk, can extend healthy lifespan in flies.

### 2.2 Neuronal knock‐down of Alk extends lifespan

**Figure 2:**
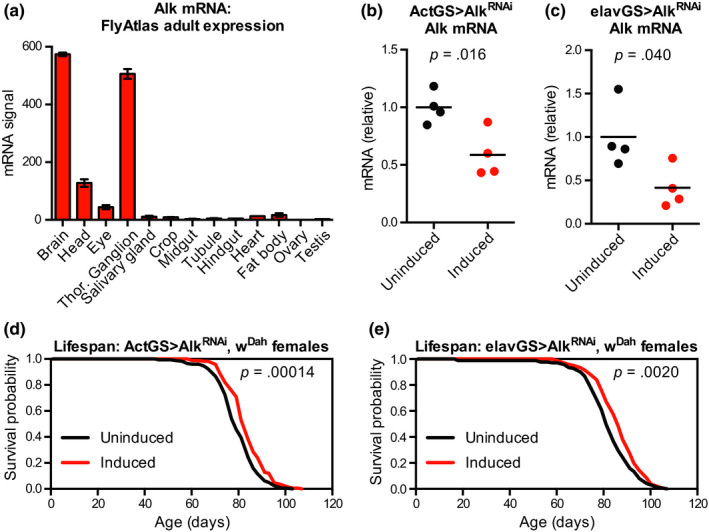
RNAi knock‐down of *Alk* ubiquitously or in neurons extends lifespan. (a) mRNA expression data from FlyAtlas showed highly enriched expression of *Alk* mRNA in nervous system tissues (brain, head, eye and thoracic‐abdominal ganglion) compared to other adult tissues. (b, c) qPCR from head RNA showed reduced *Alk* mRNA levels in (b) w^Dah^,UAS‐Alk^RNAi^/w^Dah^;Act‐GS/+; and (c) w^Dah^,UAS‐Alk^RNAi^/w^Dah^;;elav‐GS^Tricoire^/+ flies given food containing the inducing drug RU‐486 (200 μM) from 2 to 10 days of age compared to sibling flies of the same genotype treated with vehicle control food. *n* = 4 biological replicates of 6–7 heads per replicate for each condition; *p* values are from unpaired *t* tests between groups. (d) Survival curves showed extended lifespan for female w^Dah^,UAS‐Alk^RNAi^/w^Dah^;Act‐GS/+ flies treated with RU‐486 (200 μM) from 2 days of age compared to sibling flies of the same genotype treated with vehicle control food. (e) Survival curves showed extended lifespan for female w^Dah^,UAS‐Alk^RNAi^/w^Dah^;;elav‐GS^Tricoire^/+ flies treated with RU‐486 (200 μM) from 2 days of age compared to sibling flies of the same genotype treated with vehicle control food. For all survival experiments, *n > *160 deaths counted per condition; *p* values are from log‐rank tests vs. the vehicle‐treated (uninduced) group

To directly test whether reduced Alk signalling can extend lifespan, we next turned to RNAi knock‐down of Alk expression. Because Alk is necessary for proper development of both the visceral mesoderm (Englund et al., 2003) and the nervous system (Bazigou et al., 2007; Cheng et al., 2011), we restricted RNAi knock‐down of Alk to adulthood by using the GeneSwitch inducible expression system, which uses a modified GAL4 protein to drive expression of UAS transgenes only in the presence of the activating drug RU‐486 (Osterwalder, Yoon, White, & Keshishian, 2001). In adult flies, *Alk* gene expression is largely restricted to the nervous system according to FlyAtlas microarray and FlyAtlas2 RNA‐Seq data sets (Figure 2a) (Chintapalli, Wang, & Dow, 2007; Leader, Krause, Pandit, Davies, & Dow, 2018); within the nervous system, single‐cell RNA‐Seq data sets indicate that Alk gene is more highly expressed in neurons than in glial cells (Davie et al., 2018). We therefore used a ubiquitous driver, Actin5c‐GeneSwitch (Act‐GS), and an enhanced neuronal driver, elav‐GeneSwitch (elav‐GS^Tricoire^, (Latouche et al., 2007)) to reduce *Alk* gene expression in all tissues or specifically in neurons. We first confirmed by qPCR that Alk^RNAi^ driven by each GeneSwitch system significantly reduced Alk mRNA levels (Figure 2b,c, 48% decrease for Act‐GS and 58% decrease for elav‐GS).

We then assessed the lifespan of flies with ubiquitous or neuron‐specific Alk knock‐down starting in adulthood. We found that RU‐486‐treated Act‐GS > Alk^RNAi^ female flies were significantly long‐lived compared to their uninduced siblings treated with vehicle control food (Figure 2d, median lifespan + 5.8% and *p* = .00014 vs. uninduced control). This lifespan extension was reproducible in independent experiments and was not due to activation of the Act‐GS driver itself, as driver‐alone control flies showed no significant difference in lifespan when treated with RU‐486 (Figure S2a,b). We found similar effects when restricting knock‐down of Alk to neurons: RU‐486‐treated elav‐GS > Alk^RNAi^ female flies were significantly long‐lived compared to their uninduced siblings (Figure 2e, median lifespan + 6.4% and *p* = .0020 vs. uninduced control), a finding that was again reproducible in independent experiments and was not due to activation of the elav‐GS driver itself (Figure S2c,d). These results suggest that reduced Alk expression can extend healthy lifespan when restricted to adult neurons.

### 2.3 Neuronal expression of dominant‐negative Alk extends lifespan

**Figure 3:**
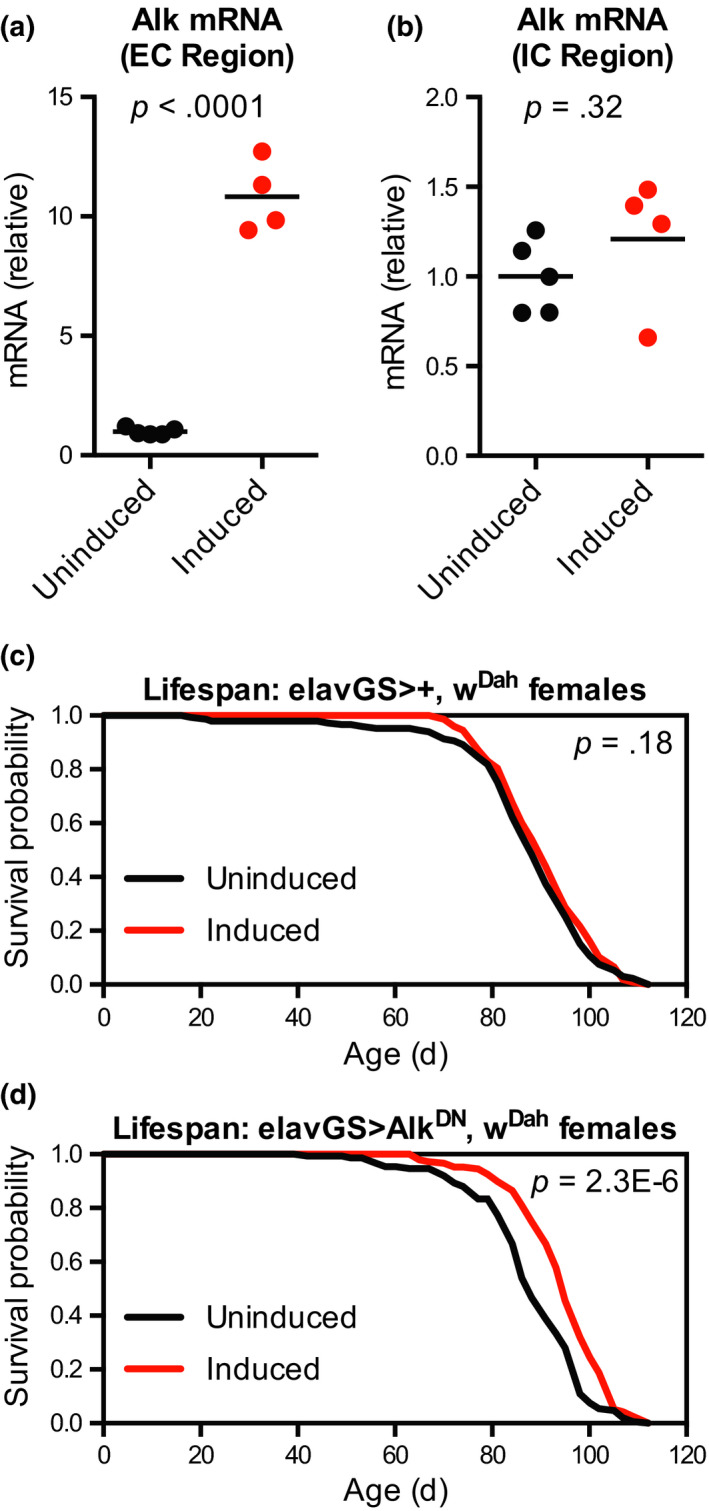
Expression of dominant‐negative *Alk* in neurons extends lifespan. (a, b) qPCR from head RNA showed increased mRNA levels for (a) the extracellular region of *Alk* but not for (b) the intracellular region of *Alk* in female w^Dah^;;UAS‐Alk^DN^/elav‐GS^301^ given food containing the inducing drug RU‐486 (200 μM) from 2 to 10 days of age compared to sibling flies of the same genotype treated with vehicle control food. *n* = 4 biological replicates of 6–7 heads per replicate for each condition; *p* values are from unpaired *t* tests between groups. (c) Survival curves showed no significant change in lifespan for female w^Dah^;;elav‐GS^301^/+ flies treated with RU‐486 (200 μM) from 2 days of age compared to sibling flies of the same genotype treated with vehicle control food. (d) Survival curves showed extended lifespan for female w^Dah^;;UAS‐Alk^DN^/elav‐GS^301^ flies treated with RU‐486 (200 μM) from 2 days of age compared to sibling flies of the same genotype treated with vehicle control food. For all survival experiments, *n* > 140 deaths counted per condition; *p* values are from log‐rank tests vs. the vehicle‐treated (uninduced) group

As an additional means of reducing Alk signalling, we turned to a UAS‐driven dominant‐negative Alk transgene (UAS‐Alk^DN^), in which only the extracellular and transmembrane domains of Alk are expressed without the intracellular signalling domains (Bazigou et al., 2007). Because of the neuron‐specific effects of Alk knock‐down described above, we restricted expression of UAS‐Alk^DN^ to adult neurons using the canonical neuronal GeneSwitch driver elav‐GS^301^ (Osterwalder et al., 2001). We first confirmed by qPCR that Alk^DN^ driven by elav‐GS significantly increased Alk mRNA levels when measured using primers directed against the extracellular region of Alk, without producing any change in Alk mRNA levels when measured using primers directed against the intracellular region (Figure 3a,b). We then assessed the lifespan of flies with neuron‐specific Alk^DN^ expression starting in adulthood. As a control, we first tested activation of the elav‐GS driver itself and found that driver‐alone control female flies showed no significant difference in lifespan when treated with RU‐486 (Figures 3c and S3a). In contrast, we found that that RU‐486‐treated elav‐GS > Alk^DN^ female flies were significantly long‐lived compared to their uninduced siblings treated with vehicle control food (Figure 3c, median lifespan + 8.0% and *p* = 2.3 × 10^−6^ vs. uninduced control). This lifespan extension was reproducible in independent experiments for female flies but not males (Figure S3b–d), consistent with our previous sexually dimorphic results for *jeb* mutant flies. Taken together with our results for RNAi knock‐down of Alk, these results suggest that reduced Alk signalling in adult neurons is sufficient to extend lifespan in females.

### 2.4 Dominant‐negative Alk expression improves locomotor behaviour, stress resistance and night sleep consolidation

**Figure 4:**
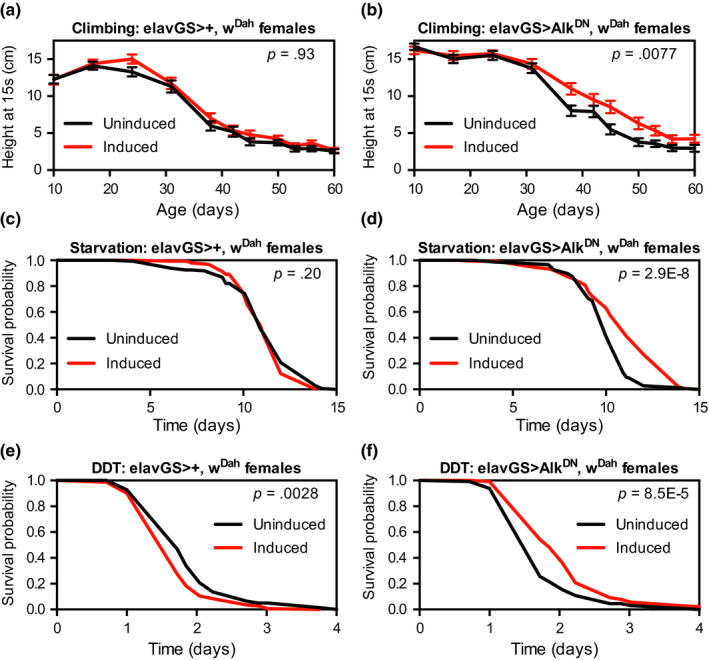
Expression of dominant‐negative *Alk* in neurons improves climbing ability, resistance to starvation, and resistance to the xenobiotic toxin DDT. (a, b) The slope of age‐related decline in climbing ability was (a) not significantly changed for female w^Dah^;;elav‐ GS^301^/+ flies and (b) significantly improved for female w^Dah^;;UAS‐Alk^DN^/elav‐GS^301^ flies treated with RU‐486 (200 μM) from 2 days of age compared to sibling flies of the same genotype treated with vehicle control food. Values shown are mean ± *SEM* from *n* > 45 individual flies for each condition and age; *p* values are from slope comparisons in a linear regression analysis. (c, d) Survival curves in starvation experiments started at 14 days of age showed (c) no significant change in survival for female w^Dah^;;elav‐GS^301^/+ flies and (d) significantly improved survival for female w^Dah^;;UAS‐Alk^DN^/elav‐GS^301^ flies treated with RU‐486 (200 μM) from 2 days of age compared to sibling flies of the same genotype treated with vehicle control food. (e, f) Survival curves in DDT (0.03%) experiments started at 14 days of age showed (c) significantly decreased survival for female w^Dah^;;elav‐GS^301^/+ flies and (d) significantly improved survival for female w^Dah^;;UAS‐Alk^DN^/elav‐GS^301^ flies treated with RU‐486 (200 μM) from 2 days of age compared to sibling flies of the same genotype treated with vehicle control food. For all survival experiments, *n* > 140 deaths counted per condition; *p* values are from log‐rank tests vs. the vehicle‐treated (uninduced) group

In addition to extending lifespan, reduced IIS and TOR signalling have been associated with preserved neuromuscular function with age (Alic et al., 2014), starvation resistance (Bjedov et al., 2010; Slack et al., 2010), resistance to xenobiotic toxins (Slack et al., 2010, 2011), and improved sleep consolidation (Metaxakis et al., 2014). To determine whether reduced Alk signalling in adult neurons would produce a similar preservation of function with age, we assessed the negative geotaxis (climbing) ability of flies with neuron‐specific Alk^DN^ expression starting in adulthood. As a control, we first tested activation of the elav‐GS driver itself and found that driver‐alone control female flies showed no significant difference in the decline of climbing ability with age when treated with RU‐486 (Figure 4a, *p* = .93 by comparison of slopes in a linear regression). In contrast, we found that that RU‐486‐treated elav‐GS > Alk^DN^ female flies had a significantly slower decline in climbing ability compared to their uninduced siblings treated with vehicle control food (Figure 4b, *p* = .0077 by comparison of slopes in a linear regression).

We next assessed resistance to starvation and xenobiotic stresses for flies with neuron‐specific Alk^DN^ expression starting in adulthood. As before, we assessed driver‐alone female flies and found no change in starvation resistance with RU‐486 treatment (Figure 4c), whereas Alk^DN^‐expressing female flies showed significant resistance to starvation (Figure 4d, median survival + 10.0% and *p* = 2.9 × 10^−8^ vs. uninduced control). We then assessed resistance to the xenobiotic toxin DDT. Here, we found that driver‐alone female flies treated with RU‐486 were significantly sensitive to DDT (Figure 4e, *p* = .0028 vs. uninduced control), but Alk^DN^‐expressing female flies nevertheless showed significant resistance to DDT (Figure 4f, median survival + 30.9% and *p* = 8.5 × 10^−5^ vs. uninduced control). In agreement with our previous results assessing lifespan, we found that male Alk^DN^‐expressing flies were not protected against either starvation or DDT stress (Figure S4). These results indicate that female flies with reduced Alk signalling in adult neurons are not only long‐lived but are also resistant to multiple stressors and have preserved neuromuscular system function with age.

**Figure 5:**
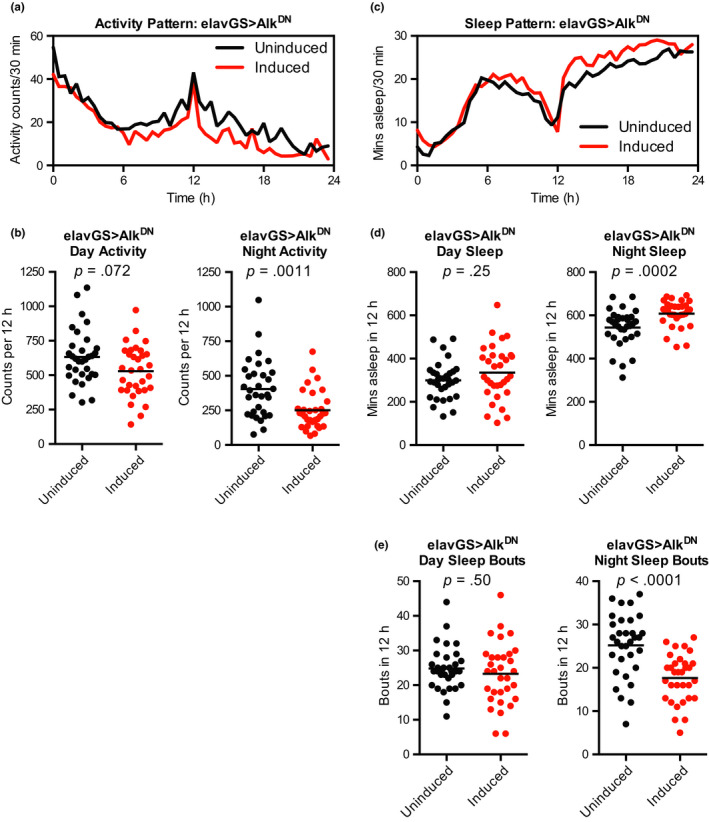
Expression of dominant‐negative *Alk* in neurons decreases night activity, increases night sleep and improves night sleep consolidation. (a) Activity traces and (b) quantification of day and night activity counts from mated female flies at 15 days of age showed significantly decreased night activity for w^Dah^;;UAS‐Alk^DN^/elav‐GS^301^ flies treated with RU‐486 (200 μM) from 2 days of age compared to sibling flies of the same genotype treated with vehicle control food. (c) Sleep traces, (d) quantification of day and night sleep, and (e) quantification of the number of day and night sleep bouts from mated female flies at 15 days of age showed significantly increased night sleep and significantly decreased numbers of night sleep bouts for w^Dah^;;UAS‐Alk^DN^/elav‐GS^301^ flies treated with RU‐486 (200 μM) from 2 days of age compared to sibling flies of the same genotype treated with vehicle control food. *n* = 32 individual flies per condition; *p* values are from Mann–Whitney tests vs. the vehicle‐treated (uninduced) group

To assess whether improvements in climbing ability were due to overall increased hyperactivity, and to measure whether Alk^DN^‐expressing flies had additional behavioural improvements, we next quantified activity and sleep patterns over the course of 24 hr (12 hr of light during the day cycle followed by 12 hr of dark in the night cycle). We found that Alk^DN^‐expressing female flies showed no evidence of hyperactivity, but instead showed significantly reduced activity during the night cycle (Figure 5a,b). We next quantified sleep, defined as continuous bouts of 5 or more minutes of inactivity, and found that Alk^DN^‐expressing flies showed significantly increased sleep amounts during the night cycle (Figure 5c,d). Next, we assessed a measure of sleep fragmentation by quantifying the number of sleep bouts. We found that Alk^DN^‐expressing flies showed significantly reduced numbers of night sleep bouts (Figure 5e), indicative of improved sleep consolidation and consistent with previous findings in long‐lived flies with reduced IIS (Metaxakis et al., 2014). Finally, we assessed driver‐alone female flies in parallel and found no change in activity or sleep patterns with RU‐486 treatment (Figure S5).

### 2.5 Dominant‐negative Alk expression reduces the expression of insulin‐like peptides

**Figure 6:**
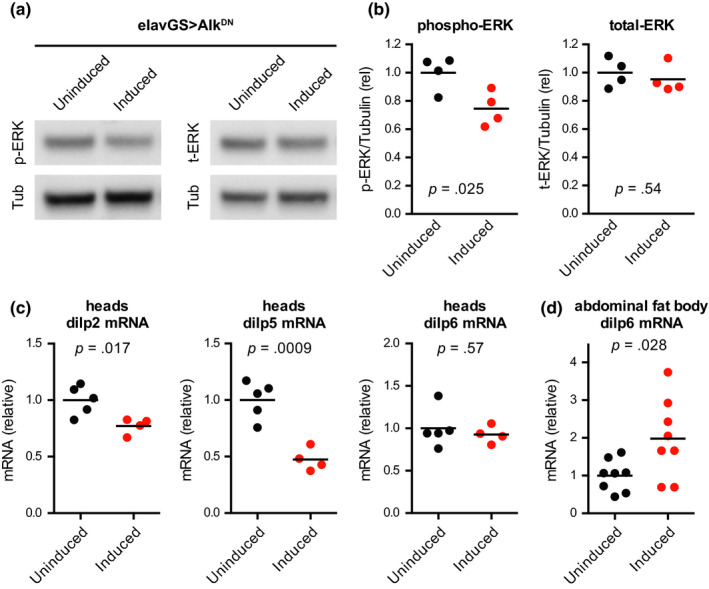
Expression of dominant‐negative *Alk* in neurons reduces Erk phosphorylation and reduces mRNA levels for *dilp2* and *dilp5*. (a, b) Western blots from head extracts showed decreased levels of phosphorylated Erk and no significant change in total Erk in female w^Dah^;;UAS‐Alk^DN^/elav‐GS^301^ flies given food containing the inducing drug RU‐486 (200 μM) from 2 to 10 days of age compared to sibling flies of the same genotype treated with vehicle control food. *n* = 4 biological replicates of 12–15 heads per replicate for each condition; *p* values are from unpaired *t* tests between groups. (c) qPCR from head RNA showed decreased mRNA levels for *dilp2* and *dilp5*, and no significant change in *dilp6*, in female w^Dah^;;UAS‐Alk^DN^/elav‐GS^301^ flies given food containing the inducing drug RU‐486 (200 μM) from 2 to 10 days of age compared to sibling flies of the same genotype treated with vehicle control food. *n* = 4–5 biological replicates of 6–7 heads per replicate for each condition; *p* values are from unpaired *t* tests between groups. (d) qPCR from abdominal fat body RNA showed increased mRNA levels for *dilp6* in female w^Dah^;;UAS‐Alk^DN^/elav‐GS^301^ flies given food containing the inducing drug RU‐486 (200 μM) from 2 to 18 days of age compared to sibling flies of the same genotype treated with vehicle control food. *n* = 8 biological replicates of four abdominal carcasses per replicate for each condition; p value is from unpaired *t* test between groups

To assess potential mechanisms by which reduced Alk signalling may promote longevity, preservation of function, and stress resistance, we first examined established signalling pathways downstream of Alk. Like other RTKs, Alk can signal through both PI3K and Ras/Erk downstream signalling pathways in species ranging from invertebrates to mammals (Palmer, Vernersson, Grabbe, & Hallberg, 2009), with Erk signalling being the best characterized pathway downstream of Alk in adult *Drosophila* (Gouzi et al., 2011). Moreover, reduced Erk signalling is one of the essential downstream mediators of enhanced longevity from reduced insulin‐like signalling in *Drosophila* (Slack et al., 2015). We therefore assessed Erk phosphorylation by Western blot from adult fly heads and found that female flies expressing Alk^DN^ starting in adulthood showed reduced levels of Erk phosphorylation with no significant change in total Erk levels (Figure 6a,b). This is consistent with previous studies observing reduced Erk phosphorylation in flies with Alk^DN^ or Alk^RNAi^ expression driven by constitutive neuronal drivers (Gouzi et al., 2011).

We next went on to assess Erk phosphorylation in both female and male head samples in parallel. Here, we again observed a significant decrease in Erk phosphorylation in Alk^DN^‐expressing females, but not in males (Figure S6a–d), consistent with our previous lifespan data showing female‐specific effects. This suggests either that males do not respond as robustly to Alk^DN^ expression, or that other upstream signalling pathways contribute more to Erk phosphorylation in males than in females. To assess additional signalling cascades downstream of Erk, we quantified Akt phosphorylation by PI3K. However, we observed no significant changes in Akt (Thr342) phosphorylation in Alk^DN^‐expressing female or male flies (Figure S6e–h), suggesting that other upstream signalling pathways may contribute more to Akt phosphorylation than Alk does.

These results prompted us to examine other downstream signalling pathways that may contribute to the lifespan extension observed with Alk inhibition. In larval *Drosophila*, Alk signalling has been found to inhibit the forkhead‐box transcription factor Foxo, thereby relieving inhibition on the expression of the *Drosophila* insulin‐like peptide 5 (dilp5) in insulin‐producing neurons (Okamoto & Nishimura, 2015). As reduced production of dilps is one of the most robust means of extending *Drosophila* lifespan (Broughton et al., 2005; Grönke et al., 2010), we asked whether we would observe reduced levels of dilp production in flies with Alk^DN^ expression in adult neurons. We found by qPCR that Alk^DN^‐expressing flies showed reduced levels of both *dilp2* and *dilp5* mRNA in head extracts (Figure 6c), whereas *dilp6*, whose expression levels are highest in glial cells and fat body within the *Drosophila* head (Chintapalli et al., 2007; Davie et al., 2018), was unaffected in the heads of Alk^DN^‐expressing flies. In the abdominal fat body, however, we found increased mRNA levels for *dilp6* in Alk^DN^‐expressing flies (Figure 6d), consistent with a previously established feedback loop in which reduced IIS increases *dilp6* transcription in the abdominal fat body, which in turn decreases *dilp2* and *dilp5* transcription in the brain (Bai, Kang, & Tatar, 2012).

To confirm the connection between reduced neuronal Alk signalling and reduced *dilp2*/*dilp5* expression, we assessed head RNA from flies with ubiquitous expression of Alk^RNAi^, where we observed a similar reduction in mRNA levels for *dilp2* and *dilp5* (Figure S7a,b). To determine whether these effects could be mediated cell‐autonomously within insulin‐producing neurons, we next drove expression of Alk^DN^ specifically in these neurons using a dilp2‐GS driver. Here, we found that Alk^DN^ expression within insulin‐producing neurons was sufficient both to reduce *dilp2* and *dilp5* expression and to produce a small but significant lifespan extension (Figure S7c–e). These results suggest that cell‐autonomous Alk modulation of *dilp2* and *dilp5* transcription within insulin‐producing neurons could provide a potential mechanism by which reduced Alk signalling can extend healthy lifespan.

### 2.6 Effects of neuronal dominant‐negative Alk expression on the abdominal fat body

To extend our findings on communication between neuronal Alk signalling and fat‐body‐derived insulin‐like peptides (Figure 6d), we next explored other phenotypes related to the fat body and lipid storage that could help explain the resistance of Alk^DN^‐expressing flies to starvation and xenobiotic toxins. We first examined lipid storage by measuring triglyceride (TAG) levels in flies expressing Alk^DN^ in neurons; however, we observed no significant change in TAG storage in these flies (Figure S8a). We went on to examine the expression of genes encoding cytochrome P450 and glutathione transferase enzymes, a subset of which are established targets of IIS and foxo in *Drosophila* (Afschar et al., 2016). We observed an unexpected significant reduction in the mRNA levels for *Cyp6a8* and a trend towards reduced mRNA levels for *Cyp6g1* in the abdominal fat bodies of flies expressing Alk^DN^ in neurons (Figure S8b,c), whereas we found no significant change in mRNA levels for *GstD1* or *GstE1* (Figure S8d,e). These results suggest that reduced neuronal Alk signalling alters the transcription of some stress response genes in distal tissues. However, it is likely that additional stress response genes and other mechanisms play important roles in the starvation and xenobiotic stress resistance of flies with reduced Alk signalling.

### 2.7 The Alk inhibitor TAE‐684 extends lifespan

**Figure 7:**
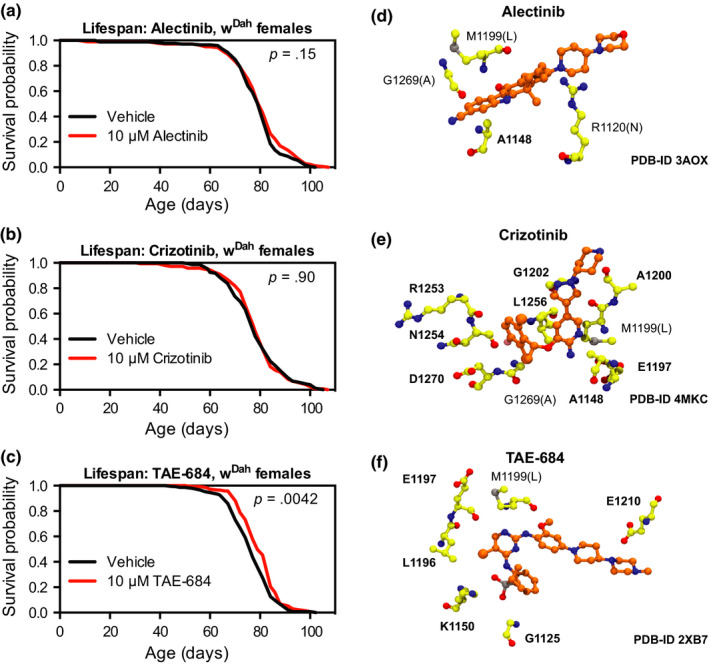
The Alk inhibitor TAE‐684, but neither alectinib nor crizotinib, extends lifespan in *Drosophila*. (a, b) Survival curves showed no significant change in lifespan for female w^Dah^ flies treated with Alectinib (10 μM) or Crizotinib (10 μM) from 2 days of age compared to sibling flies of the same genotype treated with vehicle control food. (c) Survival curves showed extended lifespan for female w^Dah^ flies treated with TAE‐684 (10 μM) from 2 days of age compared to sibling flies of the same genotype treated with vehicle control food. For all survival experiments, *n* > 135 deaths counted per condition; *p* values are from log‐rank tests vs. the vehicle‐treated group. (d, e, f) Diagrams show the amino acid residues within 3.5 A of each small molecule's binding site in published crystal structures with human Alk. Amino acids conserved between human and *Drosophila* are marked in bold; those not conserved show the *Drosophila* amino acid in parentheses

Mutations in Alk that result in its mis‐expression and/or constitutive activation have been described in a number of human cancer types, leading to the development of clinically approved small molecule Alk inhibitors (Hallberg & Palmer, 2013). In aging research, one promising direction to translation of basic research findings is the repurposing of existing drugs for use as interventions against aspects of the aging process itself (Dönertaş, Valenzuela, Partridge, & Thornton, 2018; Fuentealba et al., 2019; Ziehm et al., 2017). We therefore chose to test three small molecule Alk inhibitors for their effect on longevity: two drugs (alectinib and crizotinib) that have been clinically approved for treatment of Alk‐positive non‐small‐cell lung cancer (Kwak et al., 2010; Peters et al., 2017), and one molecule (TAE‐684) that is not yet clinically approved but has been previously used in studies of Alk function in mammalian and *Drosophila* disease models (Galkin et al., 2007; Gouzi et al., 2011, 2018). We treated flies starting at day 2 of adulthood with 100 nM, 1 µM or 10 µM of each inhibitor. For alectinib and crizotinib, we found no significant change in lifespan for wild‐type w^Dah^ female flies treated with the highest dose of 10 µM (Figure 7a,b) or lower doses of 100 nM and 1 µM (Figure S9a,b). However, for TAE‐684, we observed a significant lifespan extension for flies treated with a dose of 10 µM (Figure 7c, median survival + 3.2% and *p* = .0042 vs. vehicle‐treated control). This effect was limited to the highest dose we tested, as 100nM TAE‐684 produced no significant change in lifespan, and treatment with 1 µM TAE‐684 produced only a trend towards lifespan extension (Figure S9c, *p* = .053 vs. vehicle‐treated control).

To attempt to explain why TAE‐684, but neither alectinib nor crizotinib, could extend lifespan in *Drosophila*, we hypothesized that amino acid differences between the human and *Drosophila* Alk sequences could underlie the differential effectiveness of the small molecules, particularly if these differences occur in amino acids near the drug binding site. Crystal structures are available for alectinib, crizotinib and TAE‐684 in complex with human Alk (Bossi et al., 2010; Friboulet et al., 2014; Sakamoto et al., 2011). We used these structures to identify the amino acids within 3.5 A of each compound's binding site in the human protein and then used aligned protein sequences to identify the orthologous amino acids in the *Drosophila* protein. *Drosophila* Alk shares 34% amino acid identity overall with human Alk, with higher amino acid identity in the cytoplasmic domains (52%–58% identity) (Lorén et al., 2001). Consistent with high levels of amino acid identity, we found only a single amino acid difference among the six nearest amino acids to the binding site for TAE‐684 (83% identity). However, we found lower levels of identity for the amino acids in the vicinity of the binding sites for alectinib (25%) and crizotinib (80%) (Figure 7d–f). While these findings do not exclude the possibility that these drugs can inhibit *Drosophila* Alk, they may explain some of the differential effectiveness we saw for these three compounds in their ability to extend lifespan in *Drosophila*.

To further investigate the effects of TAE‐684, we examined signalling downstream of Alk in both heads and bodies of flies given the drug or vehicle from 2 to 10 days of age. Unlike our findings for flies with genetic inhibition of Alk signalling in neurons, we found no significant change in Erk phosphorylation in the heads of flies treated with TAE‐684 (Figure S10a,b). In the headless bodies of the same flies, we found an unexpected significant increase in Erk phosphorylation for flies treated with TAE‐684 (Figure S10c,d), suggesting either divergent signalling in different tissues, as observed for *dilp6* mRNA levels in heads vs. abdominal fat bodies (Figure 6c,d), or compensatory up‐regulation of other upstream pathways influencing Erk phosphorylation. Either way, these results suggested that TAE‐684 could have exerted its effects on Alk outside the nervous system: for example, Alk could act in the abdominal fat body where FlyAtlas data show low but present Alk expression levels (Chintapalli et al., 2007). To test whether Alk inhibition in other tissues would also be sufficient to extend lifespan, we used the S_1_106‐GS driver to express Alk^RNAi^ in the fat body and gut of adult flies. We found that Alk inhibition in these tissues was also sufficient to extend lifespan (Figure S10e), suggesting that Alk inhibition by small molecules may be able to extend lifespan by acting in non‐neuronal tissues.

## 3 DISCUSSION

We have shown that Alk inhibition, by *jeb* mutation, RNAi knock‐down of Alk in adult neurons, expression of Alk^DN^ in adult neurons, or treatment with the inhibitor TAE‐684, can extend healthy lifespan in *Drosophila*. While the lifespan extensions we observe are small (6%–8% in healthily long‐lived flies), the equivalent effects in humans would be 5–6 years of extended lifespan, and our results were highly significant and reproducible in independent experiments. In addition, we observed that inhibition of Alk signalling promoted locomotor behaviour preservation, starvation resistance, xenobiotic resistance and sleep consolidation, indicating that multiple measures of health are improved in addition to lifespan. Conversely, recent findings support a role of excessive Alk signalling in shortening healthy lifespan, as over‐expression of wild‐type human Alk leads to reduced longevity in *Drosophila* (Kim et al., 2019). Taken together, these findings lend additional support to the well‐supported theory of antagonistic pleiotropy in aging: while Alk signalling is essential for proper growth and development of the nervous system (Bazigou et al., 2007; Cheng et al., 2011; Rohrbough & Broadie, 2010), its continued activity throughout adulthood can negatively affect health and longevity.

Consistently, we have found here that interventions to inhibit Alk signalling extend healthy lifespan and stress resistance in female flies with no significant effect in males. This adds to a large body of research showing sexually dimorphic, and often female‐specific, effects of inhibition of RTK‐mediated nutrient sensing pathways. Genetic and pharmacological interventions that act on nutrient sensing pathways often show the greatest effect in females, with diminished or absent effects in males, both in flies from a variety of outbred and inbred backgrounds (Bjedov et al., 2010; Clancy et al., 2001; Regan et al., 2016; Tatar et al., 2001), and in mice from both genetically heterogeneous and homogeneous backgrounds (Harrison et al., 2009; Holzenberger et al., 2003; Selman et al., 2008, 2009). This suggests that mortality in female and male animals may be due to distinct life‐limiting pathologies in each sex (Regan & Partridge, 2013). Crucially, the effects of IIS on aging can be sexually dimorphic in humans as well: polymorphisms in IIS pathway genes have been associated with increased old‐age survival in women but not men in a Dutch population study of elderly people (van Heemst et al., 2005). Taken together, these studies consistently underscore the importance of considering whether interventions that extend healthy lifespan have sexually dimorphic effects, as appears to be the case for inhibition of Alk signalling.

One notable feature of Alk among other RTKs is its relative specificity of expression in the nervous system. While research on longevity to date has underscored an important role for the primary metabolic tissues (including the gut and adipose tissue) in the modulation of lifespan, an increasing number of studies have defined an additional role for the nervous system in modulating organism‐wide metabolism and longevity. In *Drosophila*, neuronal over‐expression of a dominant‐negative form of the insulin receptor is sufficient to extend lifespan (Augustin et al., 2018) and to maintain electrical synapse function in aging neuronal circuits (Augustin et al., 2017). Intracellular signalling pathways also play a role in neuronal modulation of lifespan: neuronal over‐expression of the nutrient sensor AMP‐activated protein kinase (AMPK), or of the autophagy‐promoting kinase Atg1, is sufficient to extend lifespan and preserve climbing ability with age in *Drosophila* (Ulgherait, Rana, Rera, Graniel, & Walker, 2014). The mechanisms underlying these neuron‐specific protective effects may be shared with the effects of inhibition of Alk signalling, as both neuronal AMPK and Atg1 over‐expression lead to a reduction in *dilp2* and *dilp5* mRNA expression (Ulgherait et al., 2014), similar to our observed effects for Alk inhibition (Figure 6). Our observed reduction in *dilp2* and *dilp5* expression is also consistent with previous studies of Alk function in the insulin‐producing neurons of the brain, where inhibition of Alk signalling can activate Foxo, which in turn negatively regulates *dilp5* transcription (Okamoto & Nishimura, 2015). Notably, however, there may be both shared and divergent mechanisms among neuronal interventions that extend healthy lifespan and stress resistance: we observe a protection against starvation stress for flies that express Alk^DN^ in neurons (Figure 4), in contrast to the sensitivity to starvation seen for flies that over‐express AMPK in neurons (Ulgherait et al., 2014). Our results also suggest that non‐cell‐autonomous effects of neuronal Alk inhibition are likely to play a role in modulating lifespan, as we observe an increase in fat‐body‐derived *dilp6* mRNA when Alk^DN^ is expressed in neurons (Figure 6). Notably, previous studies have found that over‐expression of dilp6 in the abdominal fat body can extend healthy lifespan and reduce brain mRNA levels of *dilp2* and *dilp5* (Bai et al., 2012), suggesting that a feedback loop of nutrient sensing pathways between the brain and fat body could be an important mechanism underlying healthy lifespan extension. Our results from knock‐down of Alk in the fat body (Figure S10) provide additional support for an important role of Alk signalling not only in the brain but also in the fat body.

When examining the effects of small Alk molecule inhibitors, we found no effect of either alectinib or crizotinib on *Drosophila* lifespan, and a small but significant effect of TAE‐684 (Figure 7). This is in agreement with recent studies showing no effect of crizotinib on wild‐type *Drosophila* lifespan (Kim et al., 2019) but consistent effects of TAE‐684 on developmental growth patterns and adult associative memory performance (Gouzi et al., 2011, 2018). As suggested by our structural analysis of the binding sites for each small molecule inhibitor, one potential reason for these results may be the number of conserved amino acid residues between human and *Drosophila* Alk drug binding sites for each small molecule. Other factors could also explain some of our results: for example, CNS penetrance differs among Alk inhibitors (Peters et al., 2017). However, our results also suggest that Alk inhibitors can extend lifespan without producing an observable decrease in phosphorylated Erk in head extracts, so other tissues including the fat body may play an important role (Figure S10). Moreover, drug efficacy is influenced by other factors including concentration, delivery method and food composition, so our results do not preclude the possibility that alectinib and crizotinib can effectively inhibit *Drosophila* Alk in other conditions. Taken together, our findings support careful consideration of the many factors influencing drug efficacy, including the likelihood of substrate binding in different animal species (Ziehm et al., 2017), for future pharmacological studies in model organisms.

While we observed pro‐longevity and stress‐resistance phenotypes of Alk inhibition in this study, strategies aiming to inhibit Alk are not without potential concerns. Previous studies have shown that Alk inhibition in adult animals can enhance associative memory in flies (Gouzi et al., 2011) and novel object recognition memory in mice (Bilsland et al., 2008), which can be seen as beneficial but may also suggest that Alk plays a role in suppressing inappropriate memory formation under basal conditions (Gouzi et al., 2018). Clinically, Alk inhibitors are well tolerated (Hallberg & Palmer, 2013), but unexplained side effects including visual disturbances (Kwak et al., 2010; Peters et al., 2017) suggest the need for caution before pursuing strategies requiring chronic treatment with Alk inhibitors.

Taken together, our results show that inhibition of Alk by either genetic or pharmacological means is sufficient to extend healthy lifespan and promote stress resistance in *Drosophila*. The highly conserved nature of Alk signalling among invertebrates and vertebrates lends support to further studies of Alk in mammalian aging and in models of age‐related neurodegenerative diseases. Finally, our results are encouraging for future drug repurposing studies in aging research, as existing clinically approved pharmacological inhibitors of Alk and other RTKs could have the capacity to extend healthy lifespan while potentially delaying functional decline and the onset of age‐related diseases.

## 4 METHODS

### 4.1 Fly stocks and husbandry

*Drosophila* stocks were maintained and experiments conducted at 25*°*C on a 12h:12h light:dark cycle at 60% humidity, on food containing 10% (w/v) brewer's yeast, 5% (w/v) sucrose and 1.5% (w/v) agar. The wild‐type stock *Dahomey* was collected in 1970 in Dahomey (now Benin) and has since been maintained in large population cages with overlapping generations on a 12h:12h light:dark cycle at 25ºC. The *white Dahomey* (*w^Dah^*) stock was derived by incorporation of the *w^1118^* mutation into the outbred Dahomey background by back‐crossing. All fly stocks in this study were back‐crossed for six or more generations into the outbred *w^Dah^* background. Fly stocks used were jeb^k05644^ (Bloomington #10576); Actin5c‐GS (Ford et al., 2007); elav‐GS^301^ (Osterwalder et al., 2001); elav‐GS^Tricoire^ (a stronger elav‐GS driver that in our experience produces more efficient RNAi knock‐down; a kind gift of H. Tricoire, described in (Latouche et al., 2007)); dilp2‐GS (stock #3 on the second chromosome, described in (Karpac, Hull‐Thompson, Falleur, & Jasper, 2009)); S_1_106‐GS (Roman, Endo, Zong, & Davis, 2001); UAS‐Alk^RNAi^ (Vienna GD 11446); and UAS‐Alk^DN^ (a kind gift of M. Skoulakis, described in (Bazigou et al., 2007)).

### 4.2 Addition of RU‐486 or Alk inhibitors to fly food

For all experiments involving chemical additives to fly food, the compound was dissolved in a stock solution of either ethanol or DMSO and added to the fly food while it was still liquid but had cooled to 50*°*C. The stock solution was added to the food, mixed well, dispensed into individual fly vials, and allowed to cool to room temperature overnight before storage at 4*°*C. On the day of experiments, food vials were warmed to room temperature before being used. For experiments involving the GeneSwitch system, RU‐486 (Sigma, stock solution 100 mM in ethanol) was added to the food at a final concentration of 200 µM, with 2 ml/L ethanol used as the vehicle control condition. For experiments involving Alk inhibitors, stock solutions of Alectinib (MedChem Express), Crizotinib (LKT Labs) or TAE‐684 (Generon) were prepared at 5 mM in DMSO and added to the food at the concentrations indicated in each figure. For all Alk inhibitor experimental and vehicle control conditions, DMSO was added to a final concentration of 0.2%. For all experiments, flies were allowed to develop on control food without any additives and then divided into groups to be put on vehicle‐ or drug‐containing food 2 days after emerging as adults.

### 4.3 Survival analysis

Lifespan assays were carried out as described in detail in (Piper & Partridge, 2016). From the eggs collected for each set of parental crosses, the progeny that emerged as adults within a 24 hr window were collected and allowed to mate for 48 hr, after which they were separated into single‐sex vials containing either standard food or drug‐ or vehicle‐containing food (GeneSwitch and Alk inhibitor experiments) at a density of 15 individuals per vial. For most survival assays, vials were kept in DrosoFlippers (drosoflipper.com) for ease of regular transfer to fresh vials. Flies were transferred to fresh vials three times per week, with deaths and censors scored during each transfer. Microsoft Excel (template available at http://piperlab.org/resources/) was used to calculate survival proportions.

### 4.4 Climbing (negative geotaxis) analysis

For each genotype analysed in climbing assays, five vials of control food and five vials of food containing RU‐486 (200 µM), each containing 15 flies, were housed side‐by‐side in a single DrosoFlipper. Flies were maintained as in lifespan studies and then were filmed for climbing assays once to twice per week. For climbing assays, the flies were kept in DrosoFlippers and transferred to empty vials on each side of the flipper, creating a standard vertical column 20cm in height for each set of flies. Flies were tapped to the bottom of the vials and allowed to climb upwards for 15 s before a still camera image was captured. The heights of individual flies were then assessed by manual multi‐point selection in Fiji software (Schindelin et al., 2012), with each height in pixels calibrated to a height in cm from a ruler placed next to the vials during filming.

### 4.5 Starvation and DDT stress assays

For all stress assays, flies were maintained exactly as in lifespan studies until 14 days of age. For starvation assays, flies were then transferred to vials containing 1.5% agar containing either RU‐486 (200 µM) or 2 ml/L ethanol as a control condition. Flies were then transferred to fresh vials three times per week, with deaths and censors scored once per day until day 7, then two to three times per day starting at day 7. For DDT xenobiotic stress assays, flies were transferred to vials containing 1.5% agar for 5 hr before being transferred to vials containing 0.03% DDT (Sigma) mixed into standard fly food. Deaths were scored two to three times per day without any further transfers to fresh vials.

### 4.6 Activity and Sleep analysis

Activity and sleep were assessed using the Drosophila Activity Monitor (DAM2) system and DAMSystem3 data acquisition software (Trikinetics). At 13 days of age, individual mated female flies were placed into tubes with food containing either RU‐486 (200 µM) or 2 ml/L ethanol as a control condition. These tubes were loaded into DAMs and placed into an incubator (Percival) set at 25°C, 65% humidity and a 12h:12h light:dark cycle. After two days of acclimatization, data were obtained from a 24‐hr period on the third day (beginning at the onset of lights‐on). A custom Microsoft Excel workbook (Chen, Lowe, Lamaze, Krätschmer, & Jepson, 2019) was used to calculate total activity counts per fly in the day and night periods, as well as to define sleep (five or more continuous minutes with no activity) and calculate total sleep minutes and number of sleep bouts per fly in the day and night periods.

### 4.7 PCR from genomic DNA

To identify the presence of P{lacW} in the *jeb* gene, PCR was carried out following manufacturer's instructions (All‐In Taq, highQu) on genomic DNA isolated from individual flies. Primers were designed to amplify P{lacW} independent of its insertion site (Set 1) or P{lacW} in the annotated insertion site in the *jeb* gene (Set 2). Primers sequences were as follows: LacW_Set1_for: TCGTGACTGGGAAAACCCTG; LacW_Set1_rev: TGAGGGGACGACGACAGTAT; LacW‐jeb_Set2_for: TGCTGCAAGGCGATTAAGTTG; LacW‐jeb_Set2_rev: GCGGGTAGTAGTGGCATAGT; Tub84B_for: TGGGCCCGTCTGGACCACAA; Tub84B_rev: TCGCCGTCACCGGAGTCCAT.

### 4.8 Quantitative real‐time PCR (qPCR)

Total RNA was isolated from whole flies, heads or abdomen carcasses (fat bodies) using standard TRIzol (Invitrogen) protocols. RNA samples were treated with Turbo DNAse (Invitrogen) and converted to cDNA using oligod(T) primers and Superscript II reverse transcriptase (Invitrogen). Quantitative RT–PCR was performed using Power SYBR Green PCR Master Mix (ABI) in the Quant Studio 6 Flex system. Relative quantities of transcripts were determined using the relative standard curve method normalized to *Tub84B* or *RpL9*. *Alk* primers were designed to amplify either the extracellular region (Alk‐EC primers) or intracellular region (Alk‐IC primers). Primer sequences were as follows:

jeb_for: TGCCACAAATCGAGTGTCCT; jeb_rev: CATCGCACAGCACATGATCG; Alk_EC_for: CCCTCAACCAGGCAGATGAA; Alk_EC_rev: AGGAGCATAGGTGTTCGCATT; Alk_IC_for: CTGCAATTGGTCAATGCTCTGG; Alk_IC_rev: ATTATGGCCGCCTCCTTCAG; dilp2_for: TCTGCAGTGAAAAGCTCAACGA; dilp2_rev: TCGGCACCGGGCATG; dilp5_for: GAGGCACCTTGGGCCTATTC; dilp5_rev: AAAGGAACACGATTTGCGGC; dilp6_for: AAGCTCCCAATATCAGCACCA; dilp6_rev: AGAACCATGTTTGCATGCCG; Cyp6a8_for: ATAAGGTTCGGGCTGAGGTG; Cyp6a8_rev: TGTACAGTCGCAGAGTTTCATCTA; Cyp6g1_for: ACCCTTATGCAGGAGATTGGA; Cyp6g1_rev: GCAATCGTGGCTATGCTGTC; GstD1_for: TGGTACGAGAACGCCAAGAA; GstD1_rev: TTGTCTTTGGAGACAGGCGAA; GstE1_for: GTTGGATCGCCTCAATAAGCTG; GstE1_rev: GGAGCTTCGTTGATCTCCTTG; Tub84B_for: TGGGCCCGTCTGGACCACAA; Tub84B_rev: TCGCCGTCACCGGAGTCCAT; RpL9_for: CATGATCAAGGGAGTCACGT; RpL9_rev: ATGTACTTCTCACCCAAGAAG.

### 4.9 Western blots

For protein extracts, fly heads or bodies were homogenized in RIPA buffer (New England Biolabs), with the addition of Complete Mini protease inhibitors (Roche) and PhosSTOP phosphatase inhibitors (Roche). The BCA Protein Assay Kit (Pierce) was used to calculate relative protein concentrations among the samples. Equal quantities of protein for each sample were then separated on 4%–12% NuPAGE Bis‐Tris gels (Invitrogen) and transferred to a PVDF membrane.

Membranes were blocked in 5% BSA in Tris‐buffered saline (TBS) with 0.1% Tween‐20 (TBST) for 1 hr at room temperature, after which they were probed with primary antibodies overnight at 4°C. The following primary antibodies were used: rabbit anti‐phospo‐Erk (Thr202/Tyr204) (Cell Signaling 4370, 1:2,000), rabbit anti‐Erk (Cell Signaling 4695, 1:2,000), rabbit anti‐phospho‐Akt (Thr342) (PhosphoSolutions p104‐342, 1:1,000), rabbit anti‐Akt (Cell Signaling 9272, 1:1,000), mouse anti‐Actin (Abcam ab8224, 1:10,000), mouse anti‐GAPDH (Novus NB100‐56875, 1:1,000), and mouse anti‐tubulin (Sigma T6199, 1:2,000). Membranes were then probed with secondary anti‐mouse or anti‐rabbit antibodies (Abcam ab6789 or ab6721, 1:10,000) for 1 hr at room temperature. Blots were developed using Luminata Crescendo or Forte (Millipore) and the ImageQuant LAS 4000 system. Densitometric analysis of blot images was carried out using Fiji software (Schindelin et al., 2012).

### 4.10 Triglyceride (TAG) Assays

Triglyceride Infinity Reagent (Thermo Scientific) was used to measure total triglyceride content from homogenates of whole single flies. Values were normalized to the protein content per fly as measured by the Pierce BCA Protein Assay Kit (Thermo Scientific).

### 4.11 Conservation of binding sites for Alk inhibitors

Crystallographic structures of Alk in complex with alectinib, crizotinib and TAE‐684 were downloaded from the Protein Data Bank (http://www.rcsb.org). Amino acid residues within 3.5 A of the Alk inhibitors were visualized using the software VMD 1.9.4 (Humphrey, Dalke, & Schulten, 1996). The sequence of the human Alk (UniProt ID Q9UM73) and the Drosophila Alk (UniProt ID Q7KJ08) were aligned using the UniProt Align function, and the binding residues were manually located in the sequence alignment. The sequence identity of the binding site was calculated as the percentage of residues in the human Alk binding site conserved in the *Drosophila* orthologue.

### 4.12 Statistical analysis

Statistical analysis was carried out in Microsoft Excel, GraphPad Prism 6.0a, or R using the “survival” package (Terry Therneau, https://CRAN.R-project.org/package=survival). The statistical test used for each experiment is indicated in the figure legend. Log‐rank tests on lifespan data were performed in Microsoft Excel (template available at http://piperlab.org/resources/ ) or in R. Linear regression analysis was performed on climbing data, and ANOVA, Mann–Whitney and *t* test analyses were performed on activity, sleep, qPCR and Western blot data in GraphPad Prism. For all statistical tests, *p* < .05 was considered significant.

## CONFLICT OF INTEREST

The authors declare no conflict of interest.

## **AUTHOR** **CONTRIBUTIONS**

N.S.W. and L.P. conceived the study; N.S.W., B.A., M.C.D., L.J.M, A.R., A.J.D., K.H.C.L. and S.P. designed the methodology; N.S.W., B.A., M.C.D., L.J.M, A.R., A.J.D., K.H.C.L. and S.P. investigated the study; N.S.W., B.A., M.C.D., L.J.M, A.R., A.J.D., K.H.C.L., S.P. and M.F. involved in formal analysis; N.S.W. wrote the original draft of the manuscript; N.S.W. and L.P. wrote, reviewed and edited the manuscript; N.S.W. and M.F. visualized the study; N.S.W., N.A. and L.P. supervised the study; N.S.W. and L.P. involved in project administration; N.S.W., N.A. and L.P involved in funding acquisition.

## Supporting information

Fig S1‐S10

## Data Availability

The raw data supporting this study are available in Mendeley Data at http://dx.doi.org/10.17632/k392rcs98d.1

## References

[acel13137-bib-0001] Afschar, S. , Toivonen, J. M. , Hoffmann, J. M. , Tain, L. S. , Wieser, D. , Finlayson, A. J. … Partridge, L. (2016). Nuclear hormone receptor DHR96 mediates the resistance to xenobiotics but not the increased lifespan of insulin‐mutant Drosophila. Proceedings of the National Academy of Sciences of the United States of America, 113(5), 1321–1326. 10.1073/pnas.1515137113 26787908 PMC4747718

[acel13137-bib-0002] Alic, N. , & Partridge, L. (2011). Death and dessert: Nutrient signalling pathways and ageing. Current Opinion in Cell Biology, 23(6), 738–743. 10.1016/j.ceb.2011.07.006 21835601 PMC4335171

[acel13137-bib-0003] Alic, N. , Tullet, J. M. , Niccoli, T. , Broughton, S. , Hoddinott, M. P. , Slack, C. , … Partridge, L. (2014). Cell‐nonautonomous effects of dFOXO/DAF‐16 in aging. Cell Reports, 6(4), 608–616. 10.1016/j.celrep.2014.01.015 24508462 PMC3969275

[acel13137-bib-0004] Augustin, H. , McGourty, K. , Allen, M. J. , Adcott, J. , Wong, C. T. , Boucrot, E. , & Partridge, L. (2018). Impact of insulin signaling and proteasomal activity on physiological output of a neuronal circuit in aging Drosophila melanogaster. Neurobiology of Aging, 66, 149–157. 10.1016/j.neurobiolaging.2018.02.027 29579685 PMC5933513

[acel13137-bib-0005] Augustin, H. , McGourty, K. , Allen, M. J. , Madem, S. K. , Adcott, J. , Kerr, F. , … Partridge, L. (2017). Reduced insulin signaling maintains electrical transmission in a neural circuit in aging flies. PLoS Biology, 15(9), e2001655 10.1371/journal.pbio.2001655 28902870 PMC5597081

[acel13137-bib-0006] Bai, H. , Kang, P. , & Tatar, M. (2012). Drosophila insulin‐like peptide‐6 (dilp6) expression from fat body extends lifespan and represses secretion of Drosophila insulin‐like peptide‐2 from the brain. Aging Cell, 11(6), 978–985. 10.1111/acel.12000 22935001 PMC3500397

[acel13137-bib-0007] Bazigou, E. , Apitz, H. , Johansson, J. , Lorén, C. E. , Hirst, E. M. A. , Chen, P.‐L. , … Salecker, I. (2007). Anterograde Jelly belly and Alk receptor tyrosine kinase signaling mediates retinal axon targeting in Drosophila. Cell, 128(5), 961–975. 10.1016/j.cell.2007.02.024 17350579

[acel13137-bib-0008] Bilsland, J. G. , Wheeldon, A. , Mead, A. , Znamenskiy, P. , Almond, S. , Waters, K. A. , … Munoz‐Sanjuan, I. (2008). Behavioral and neurochemical alterations in mice deficient in anaplastic lymphoma kinase suggest therapeutic potential for psychiatric indications. Neuropsychopharmacology, 33(3), 685–700. 10.1038/sj.npp.1301446 17487225

[acel13137-bib-0009] Bjedov, I. , Toivonen, J. M. , Kerr, F. , Slack, C. , Jacobson, J. , Foley, A. , & Partridge, L. (2010). Mechanisms of life span extension by rapamycin in the fruit fly Drosophila melanogaster. Cell Metabolism, 11(1), 35–46. 10.1016/j.cmet.2009.11.010 20074526 PMC2824086

[acel13137-bib-0010] Bossi, R. T. , Saccardo, M. B. , Ardini, E. , Menichincheri, M. , Rusconi, L. , Magnaghi, P. , … Bertrand, J. A. (2010). Crystal structures of anaplastic lymphoma kinase in complex with ATP competitive inhibitors. Biochemistry, 49(32), 6813–6825. 10.1021/bi1005514 20695522

[acel13137-bib-0011] Broughton, S. J. , Piper, M. D. W. , Ikeya, T. , Bass, T. M. , Jacobson, J. , Driege, Y. , … Partridge, L. (2005). Longer lifespan, altered metabolism, and stress resistance in Drosophila from ablation of cells making insulin‐like ligands. Proceedings of the National Academy of Sciences of the United States of America, 102(8), 3105–3110. 10.1073/pnas.0405775102 15708981 PMC549445

[acel13137-bib-0012] Chen, K.‐F. , Lowe, S. , Lamaze, A. , Krätschmer, P. , & Jepson, J. (2019). Neurocalcin regulates nighttime sleep and arousal in Drosophila. eLife, 8, 1717 10.7554/eLife.38114 PMC6415939 30865587

[acel13137-bib-0013] Cheng, L. Y. , Bailey, A. P. , Leevers, S. J. , Ragan, T. J. , Driscoll, P. C. , & Gould, A. P. (2011). Anaplastic lymphoma kinase spares organ growth during nutrient restriction in drosophila. Cell, 146(3), 435–447. 10.1016/j.cell.2011.06.040 21816278

[acel13137-bib-0014] Chintapalli, V. R. , Wang, J. , & Dow, J. A. T. (2007). Using FlyAtlas to identify better Drosophila melanogaster models of human disease. Nature Genetics, 39(6), 715–720. 10.1038/ng2049 17534367

[acel13137-bib-0015] Clancy, D. J. , Gems, D. , Harshman, L. G. , Oldham, S. , Stocker, H. , Hafen, E. , … Partridge, L. (2001). Extension of life‐span by loss of CHICO, a Drosophila insulin receptor substrate protein. Science, 292(5514), 104–106. 10.1126/science.1057991 11292874

[acel13137-bib-0016] Davie, K. , Janssens, J. , Koldere, D. , De Waegeneer, M. , Pech, U. , Kreft, Ł. , … Aerts, S. (2018). A single‐cell transcriptome atlas of the aging drosophila brain. Cell, 174(4), 982–998.e20. 10.1016/j.cell.2018.05.057 29909982 PMC6086935

[acel13137-bib-0017] Dönertaş, H. M. , Valenzuela, M. F. , Partridge, L. , & Thornton, J. M. (2018). Gene expression‐based drug repurposing to target ageing. Aging Cell, 8(9), e12819 10.1111/acel.12819 PMC6156541 29959820

[acel13137-bib-0018] Englund, C. , Lorén, C. E. , Grabbe, C. , Varshney, G. K. , Deleuil, F. , Hallberg, B. , & Palmer, R. H. (2003). Jeb signals through the Alk receptor tyrosine kinase to drive visceral muscle fusion. Nature, 425(6957), 512–516. 10.1038/nature01950 14523447

[acel13137-bib-0019] Fadeev, A. , Mendoza‐Garcia, P. , Irion, U. , Guan, J. , Pfeifer, K. , Wiessner, S. , … Palmer, R. H. (2018). ALKALs are in vivo ligands for ALK family receptor tyrosine kinases in the neural crest and derived cells. Proceedings of the National Academy of Sciences of the United States of America, 115(4), E630–E638. 10.1073/pnas.1719137115 29317532 PMC5789956

[acel13137-bib-0020] Flachsbart, F. , Caliebe, A. , Kleindorp, R. , Blanché, H. , von Eller‐Eberstein, H. , Nikolaus, S. , … Nebel, A. (2009). Association of FOXO3A variation with human longevity confirmed in German centenarians. Proceedings of the National Academy of Sciences of the United States of America, 106(8), 2700–2705. 10.1073/pnas.0809594106 19196970 PMC2650329

[acel13137-bib-0021] Fontana, L. , Partridge, L. , & Longo, V. D. (2010). Extending healthy life span–from yeast to humans. Science, 328(5976), 321–326. 10.1126/science.1172539 20395504 PMC3607354

[acel13137-bib-0022] Ford, D. , Hoe, N. , Landis, G. , Tozer, K. , Luu, A. , Bhole, D. , … Tower, J. (2007). Alteration of Drosophila life span using conditional, tissue‐specific expression of transgenes triggered by doxycyline or RU486/Mifepristone. Experimental Gerontology, 42(6), 483–497. 10.1016/j.exger.2007.01.004 17349761 PMC1992522

[acel13137-bib-0023] Friboulet, L. , Li, N. , Katayama, R. , Lee, C. C. , Gainor, J. F. , Crystal, A. S. , … Engelman, J. A. (2014). The ALK inhibitor ceritinib overcomes crizotinib resistance in non‐small cell lung cancer. Cancer Discovery, 4(6), 662–673. 10.1158/2159-8290.CD-13-0846 24675041 PMC4068971

[acel13137-bib-0024] Fuentealba, M. , Dönertaş, H. M. , Williams, R. , Labbadia, J. , Thornton, J. M. , & Partridge, L. (2019). Using the drug‐protein interactome to identify anti‐ageing compounds for humans. PLoS Computational Biology, 15(1), e1006639 10.1371/journal.pcbi.1006639 30625143 PMC6342327

[acel13137-bib-0025] Galkin, A. V. , Melnick, J. S. , Kim, S. , Hood, T. L. , Li, N. , Li, L. , … Warmuth, M. (2007). Identification of NVP‐TAE684, a potent, selective, and efficacious inhibitor of NPM‐ALK. Proceedings of the National Academy of Sciences of the United States of America, 104(1), 270–275. 10.1073/pnas.0609412103 17185414 PMC1765448

[acel13137-bib-0026] Giannakou, M. E. , Goss, M. , Jünger, M. A. , Hafen, E. , Leevers, S. J. , & Partridge, L. (2004). Long‐lived Drosophila with overexpressed dFOXO in adult fat body. Science, 305(5682), 361 10.1126/science.1098219 15192154

[acel13137-bib-0027] Gouzi, J. Y. , Bouraimi, M. , Roussou, I. G. , Moressis, A. , & Skoulakis, E. M. C. (2018). The drosophila receptor tyrosine kinase Alk constrains long‐term memory formation. Journal of Neuroscience, 38(35), 7701–7712. 10.1523/JNEUROSCI.0784-18.2018 30030398 PMC6705970

[acel13137-bib-0028] Gouzi, J. Y. , Moressis, A. , Walker, J. A. , Apostolopoulou, A. A. , Palmer, R. H. , Bernards, A. , & Skoulakis, E. M. C. (2011). The receptor tyrosine kinase Alk controls neurofibromin functions in Drosophila growth and learning. PLoS Genetics, 7(9), e1002281 10.1371/journal.pgen.1002281 21949657 PMC3174217

[acel13137-bib-0029] Grönke, S. , Clarke, D.‐F. , Broughton, S. , Andrews, T. D. , & Partridge, L. (2010). Molecular evolution and functional characterization of Drosophila insulin‐like peptides. PLoS Genetics, 6(2), e1000857 10.1371/journal.pgen.1000857.t001 20195512 PMC2829060

[acel13137-bib-0030] Guan, J. , Umapathy, G. , Yamazaki, Y. , Wolfstetter, G. , Mendoza, P. , Pfeifer, K. , … Palmer, R. H. (2015). FAM150A and FAM150B are activating ligands for anaplastic lymphoma kinase. eLife, 4, e09811 10.7554/eLife.09811 26418745 PMC4658194

[acel13137-bib-0031] Hallberg, B. , & Palmer, R. H. (2013). Mechanistic insight into ALK receptor tyrosine kinase in human cancer biology. Nature Reviews Cancer, 13(10), 685–700. 10.1038/nrc3580 24060861

[acel13137-bib-0032] Harrison, D. E. , Strong, R. , Sharp, Z. D. , Nelson, J. F. , Astle, C. M. , Flurkey, K. , … Miller, R. A. (2009). Rapamycin fed late in life extends lifespan in genetically heterogeneous mice. Nature, 460(7253), 392–395. 10.1038/nature08221 19587680 PMC2786175

[acel13137-bib-0033] Holzenberger, M. , Dupont, J. , Ducos, B. , Leneuve, P. , Géloën, A. , Even, P. C. , … Le Bouc, Y. (2003). IGF‐1 receptor regulates lifespan and resistance to oxidative stress in mice. Nature, 421(6919), 182–187. 10.1038/nature01298 12483226

[acel13137-bib-0034] Humphrey, W. , Dalke, A. , & Schulten, K. (1996). VMD: Visual molecular dynamics. Journal of Molecular Graphics, 14(1), 33–38. 10.1016/0263-7855(96)00018-5 8744570

[acel13137-bib-0035] Hwangbo, D. S. , Gershman, B. , Gersham, B. , Tu, M.‐P. , Palmer, M. , & Tatar, M. (2004). Drosophila dFOXO controls lifespan and regulates insulin signalling in brain and fat body. Nature, 429(6991), 562–566. 10.1038/nature02549 15175753

[acel13137-bib-0036] Karpac, J. , Hull‐Thompson, J. , Falleur, M. , & Jasper, H. (2009). JNK signaling in insulin‐producing cells is required for adaptive responses to stress in Drosophila. Aging Cell, 8(3), 288–295. 10.1111/j.1474-9726.2009.00476.x 19627268 PMC2727449

[acel13137-bib-0037] Kim, Y. J. , Cho, A.‐R. , Sul, H. J. , Kim, B. , Kim, A.‐Y. , Kim, H. S. et al. (2019). The effects of crizotinib in a transgenic Drosophila model expressing the human TPM4‐ALK fusion gene or TPM4. Biology Open, 8(7), bio044362 10.1242/bio.044362 31278140 PMC6679403

[acel13137-bib-0038] Kontis, V. , Bennett, J. E. , Mathers, C. D. , Li, G. , Foreman, K. , & Ezzati, M. (2017). Future life expectancy in 35 industrialised countries: Projections with a Bayesian model ensemble. Lancet, 389(10076), 1323–1335. 10.1016/S0140-6736(16)32381-9 28236464 PMC5387671

[acel13137-bib-0039] Kwak, E. L. , Bang, Y.‐J. , Camidge, D. R. , Shaw, A. T. , Solomon, B. , Maki, R. G. , … Iafrate, A. J. (2010). Anaplastic lymphoma kinase inhibition in non‐small‐cell lung cancer. The New England Journal of Medicine, 363(18), 1693–1703. 10.1056/NEJMoa1006448 20979469 PMC3014291

[acel13137-bib-0040] Latouche, M. , Lasbleiz, C. , Martin, E. , Monnier, V. , Debeir, T. , Mouatt‐Prigent, A. , … Tricoire, H. (2007). A conditional pan‐neuronal Drosophila model of spinocerebellar ataxia 7 with a reversible adult phenotype suitable for identifying modifier genes. Journal of Neuroscience, 27(10), 2483–2492. 10.1523/JNEUROSCI.5453-06.2007 17344386 PMC6672519

[acel13137-bib-0041] Leader, D. P. , Krause, S. A. , Pandit, A. , Davies, S. A. , & Dow, J. A. T. (2018). FlyAtlas 2: A new version of the Drosophila melanogaster expression atlas with RNA‐Seq, miRNA‐Seq and sex‐specific data. Nucleic Acids Research, 46(D1), D809–D815. 10.1093/nar/gkx976 29069479 PMC5753349

[acel13137-bib-0042] Lemmon, M. A. , & Schlessinger, J. (2010). Cell signaling by receptor tyrosine kinases. Cell, 141(7), 1117–1134. 10.1016/j.cell.2010.06.011 20602996 PMC2914105

[acel13137-bib-0043] López‐Otín, C. , Blasco, M. A. , Partridge, L. , Serrano, M. , & Kroemer, G. (2013). The hallmarks of aging. Cell, 153(6), 1194–1217. 10.1016/j.cell.2013.05.039 23746838 PMC3836174

[acel13137-bib-0044] Loren, C. E. , Scully, A. , Grabbe, C. , Edeen, P. T. , Thomas, J. , McKeown, M. , … Palmer, R. H. (2001). Identification and characterization of DAlk: A novel Drosophila melanogaster RTK which drives ERK activation in vivo. Genes to Cells, 6(6), 531–544. 10.1046/j.1365-2443.2001.00440.x 11442633 PMC1975818

[acel13137-bib-0045] Metaxakis, A. , Tain, L. S. , Grönke, S. , Hendrich, O. , Hinze, Y. , Birras, U. , & Partridge, L. (2014). Lowered insulin signalling ameliorates age‐related sleep fragmentation in Drosophila. PLoS Biology, 12(4), e1001824 10.1371/journal.pbio.1001824 24690889 PMC3972082

[acel13137-bib-0046] Oeppen, J. , & Vaupel, J. W. (2002). Demography. Broken limits to life expectancy. Science, 296(5570), 1029–1031. 10.1126/science.1069675 12004104

[acel13137-bib-0047] Okamoto, N. , & Nishimura, T. (2015). Signaling from glia and cholinergic neurons controls nutrient‐dependent production of an insulin‐like peptide for drosophila body growth. Developmental Cell, 35(3), 295–310. 10.1016/j.devcel.2015.10.003 26555050

[acel13137-bib-0048] Osterwalder, T. , Yoon, K. S. , White, B. H. , & Keshishian, H. (2001). A conditional tissue‐specific transgene expression system using inducible GAL4. Proceedings of the National Academy of Sciences of the United States of America, 98(22), 12596–12601. 10.1073/pnas.221303298 11675495 PMC60099

[acel13137-bib-0049] Palmer, R. H. , Vernersson, E. , Grabbe, C. , & Hallberg, B. (2009). Anaplastic lymphoma kinase: Signalling in development and disease. Biochemical Journal, 420(3), 345–361. 10.1042/BJ20090387 19459784 PMC2708929

[acel13137-bib-0050] Peters, S. , Camidge, D. R. , Shaw, A. T. , Gadgeel, S. , Ahn, J. S. , Kim, D.‐W. , … ALEX Trial Investigators (2017). Alectinib versus crizotinib in untreated ALK‐positive non‐small‐cell lung cancer. The New England Journal of Medicine, 377(9), 829–838. 10.1056/NEJMoa1704795 28586279

[acel13137-bib-0051] Piper, M. D. W. , & Partridge, L. (2016). Protocols to study aging in drosophila. Methods in Molecular Biology, 1478, 291–302. 10.1007/978-1-4939-6371-3_18 27730590 PMC5507281

[acel13137-bib-0052] Regan, J. C. , Khericha, M. , Dobson, A. J. , Bolukbasi, E. , Rattanavirotkul, N. , & Partridge, L. (2016). Sex difference in pathology of the ageing gut mediates the greater response of female lifespan to dietary restriction. eLife, 5, 10.7554/eLife.10956 PMC4805549 26878754

[acel13137-bib-0053] Regan, J. C. , & Partridge, L. (2013). Gender and longevity: Why do men die earlier than women? Comparative and experimental evidence. Best Practice & Research Clinical Endocrinology & Metabolism, 27(4), 467–479. 10.1016/j.beem.2013.05.016 24054925

[acel13137-bib-0054] Rohrbough, J. , & Broadie, K. (2010). Anterograde Jelly belly ligand to Alk receptor signaling at developing synapses is regulated by Mind the gap. Development (Cambridge, England), 137(20), 3523–3533. 10.1242/dev.047878 PMC2947762 20876658

[acel13137-bib-0055] Roman, G. , Endo, K. , Zong, L. , & Davis, R. L. (2001). P[Switch], a system for spatial and temporal control of gene expression in Drosophila melanogaster. Proceedings of the National Academy of Sciences of the United States of America, 98(22), 12602–12607. 10.1073/pnas.221303998 11675496 PMC60100

[acel13137-bib-0056] Sakamoto, H. , Tsukaguchi, T. , Hiroshima, S. , Kodama, T. , Kobayashi, T. , Fukami, T. A. , … Aoki, Y. (2011). CH5424802, a selective ALK inhibitor capable of blocking the resistant gatekeeper mutant. Cancer Cell, 19(5), 679–690. 10.1016/j.ccr.2011.04.004 21575866

[acel13137-bib-0057] Schindelin, J. , Arganda‐Carreras, I. , Frise, E. , Kaynig, V. , Longair, M. , Pietzsch, T. , … Cardona, A. (2012). Fiji: An open‐source platform for biological‐image analysis. Nature Methods, 9(7), 676–682. 10.1038/nmeth.2019 22743772 PMC3855844

[acel13137-bib-0058] Selman, C. , Lingard, S. , Choudhury, A. I. , Batterham, R. L. , Claret, M. , Clements, M. , … Withers, D. J. (2008). Evidence for lifespan extension and delayed age‐related biomarkers in insulin receptor substrate 1 null mice. The FASEB Journal, 22(3), 807–818. 10.1096/fj.07-9261com 17928362

[acel13137-bib-0059] Selman, C. , Tullet, J. M. A. , Wieser, D. , Irvine, E. , Lingard, S. J. , Choudhury, A. I. , … Withers, D. J. (2009). Ribosomal protein S6 kinase 1 signaling regulates mammalian life span. Science, 326(5949), 140–144. 10.1126/science.1177221 19797661 PMC4954603

[acel13137-bib-0060] Slack, C. , Alic, N. , Foley, A. , Cabecinha, M. , Hoddinott, M. P. , & Partridge, L. (2015). The Ras‐Erk‐ETS‐signaling pathway is a drug target for longevity. Cell, 162(1), 72–83. 10.1016/j.cell.2015.06.023 26119340 PMC4518474

[acel13137-bib-0061] Slack, C. , Giannakou, M. E. , Foley, A. , Goss, M. , & Partridge, L. (2011). dFOXO‐independent effects of reduced insulin‐like signaling in Drosophila. Aging Cell, 10(5), 735–748. 10.1111/j.1474-9726.2011.00707.x 21443682 PMC3193374

[acel13137-bib-0062] Slack, C. , Werz, C. , Wieser, D. , Alic, N. , Foley, A. , Stocker, H. et al. (2010). Regulation of lifespan, metabolism, and stress responses by the Drosophila SH2B protein, Lnk. Plos Genetics, 6(3), e1000881 10.1371/journal.pgen.1000881.g006 20333234 PMC2841611

[acel13137-bib-0063] Sopko, R. , & Perrimon, N. (2013). Receptor tyrosine kinases in drosophila development. Cold Spring Harbor Perspectives in Biology, 5(6), a009050–a009050. 10.1101/cshperspect.a009050 23732470 PMC3660834

[acel13137-bib-0064] Suh, Y. , Atzmon, G. , Cho, M.‐O. , Hwang, D. , Liu, B. , Leahy, D. J. , … Cohen, P. (2008). Functionally significant insulin‐like growth factor I receptor mutations in centenarians. Proceedings of the National Academy of Sciences of the United States of America, 105(9), 3438–3442. 10.1073/pnas.0705467105 18316725 PMC2265137

[acel13137-bib-0065] Tatar, M. , Kopelman, A. , Epstein, D. , Tu, M. P. , Yin, C. M. , & Garofalo, R. S. (2001). A mutant Drosophila insulin receptor homolog that extends life‐span and impairs neuroendocrine function. Science, 292(5514), 107–110. 10.1126/science.1057987 11292875

[acel13137-bib-0066] Ulgherait, M. , Rana, A. , Rera, M. , Graniel, J. , & Walker, D. W. (2014). AMPK modulates tissue and organismal aging in a non‐cell‐autonomous manner. Cell Reports, 8(6), 1767–1780. 10.1016/j.celrep.2014.08.006 25199830 PMC4177313

[acel13137-bib-0067] Van Heemst, D. , Beekman, M. , Mooijaart, S. P. , Heijmans, B. T. , Brandt, B. W. , Zwaan, B. J. , … Westendorp, R. G. J. (2005). Reduced insulin/IGF‐1 signalling and human longevity. Aging Cell, 4(2), 79–85. 10.1111/j.1474-9728.2005.00148.x 15771611

[acel13137-bib-0068] Weiss, J. B. , Suyama, K. L. , Lee, H. H. , & Scott, M. P. (2001). Jelly belly: A Drosophila LDL receptor repeat‐containing signal required for mesoderm migration and differentiation. Cell, 107(3), 387–398. 10.1016/S0092-8674(01)00540-2 11701128

[acel13137-bib-0069] Willcox, B. J. , Donlon, T. A. , He, Q. , Chen, R. , Grove, J. S. , Yano, K. , … Curb, J. D. (2008). FOXO3A genotype is strongly associated with human longevity. Proceedings of the National Academy of Sciences of the United States of America, 105(37), 13987–13992. 10.1073/pnas.0801030105 18765803 PMC2544566

[acel13137-bib-0070] Yao, S. , Cheng, M. , Zhang, Q. , Wasik, M. , Kelsh, R. , & Winkler, C. (2013). Anaplastic lymphoma kinase is required for neurogenesis in the developing central nervous system of zebrafish. PLoS ONE, 8(5), e63757 10.1371/journal.pone.0063757 23667670 PMC3648509

[acel13137-bib-0071] Ziehm, M. , Kaur, S. , Ivanov, D. K. , Ballester, P. J. , Marcus, D. , Partridge, L. , & Thornton, J. M. (2017). Drug repurposing for aging research using model organisms. Aging Cell, 16(5), 1006–1015. 10.1111/acel.12626 28620943 PMC5595691

