# Supplementary material for: The neuronal receptor tyrosine kinase Alk is a target for longevity
Source: Aging Cell. 2020 Apr 15;19(5):e13137. doi: 10.1111/acel.13137 (PMC7253064; doi:10.1111/acel.13137)
Supplement: Supplementary file 1 — Fig S1‐S10 [file ACEL-19-e13137-s001.pdf]

Supplementary Figures for

The neuronal receptor tyrosine kinase Alk is a target for longevity

Nathaniel S. Woodling[1], Benjamin Aleyakpo[1], Miranda Claire Dyson[1], Lucy J. Minkley[1], Arjunan Rajasingam[1], Adam J. Dobson[1,3], Kristie H. C. Leung[1], Simona Pomposova[1], Matías Fuentealba[1], Nazif Alic[1], and Linda Partridge[1,2]

[1] Institute of Healthy Ageing and Department of Genetics, Evolution and Environment, University College London, Darwin Building, Gower Street, London WC1E 6BT, United Kingdom

[2] Max Planck Institute for Biology of Ageing, Joseph-Stelzmann-Strasse 9b, 50931 Cologne, Germany

[3] Current Address: Institute of Molecular, Cell and Systems Biology, College of Medical, Veterinary and Life Sciences, Davidson Building, University of Glasgow, Glasgow, G12 8QQ, United Kingdom

Correspondence should be addressed to:

Linda Partridge
Institute of Healthy Ageing
University College London
Tel: (+44) 020 7679 4380
l.partridge@ucl.ac.uk

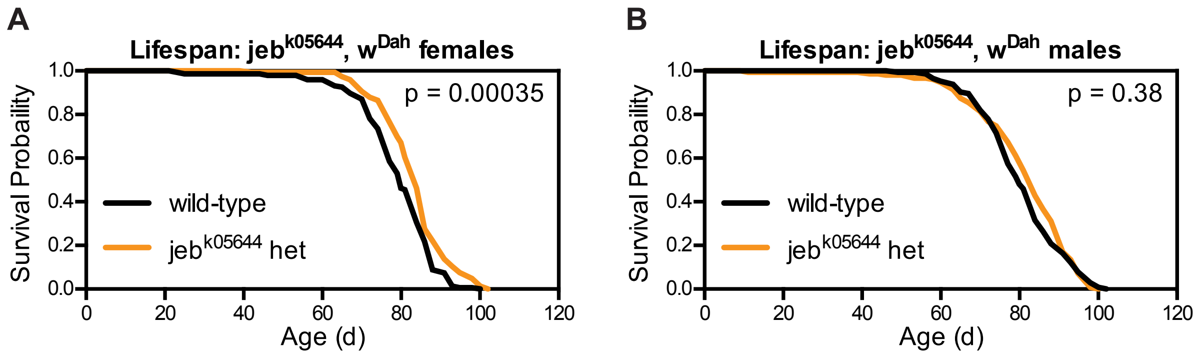

Supplementary Figure 1. Heterozygous loss of *jeb* reproducibly extends lifespan in female but not male flies. (**A**) Survival curves showed extended lifespan for female w^{Dah};jeb^{k05644}/+; flies compared to their w^{Dah};+/+; siblings. (**B**) Survival curves showed no significant change in lifespan for male w^{Dah};jeb^{k05644}/+; flies compared to their w^{Dah};+/+; siblings. For all survival experiments, n>145 deaths counted per condition; p values are from log-rank tests versus the wild-type condition.

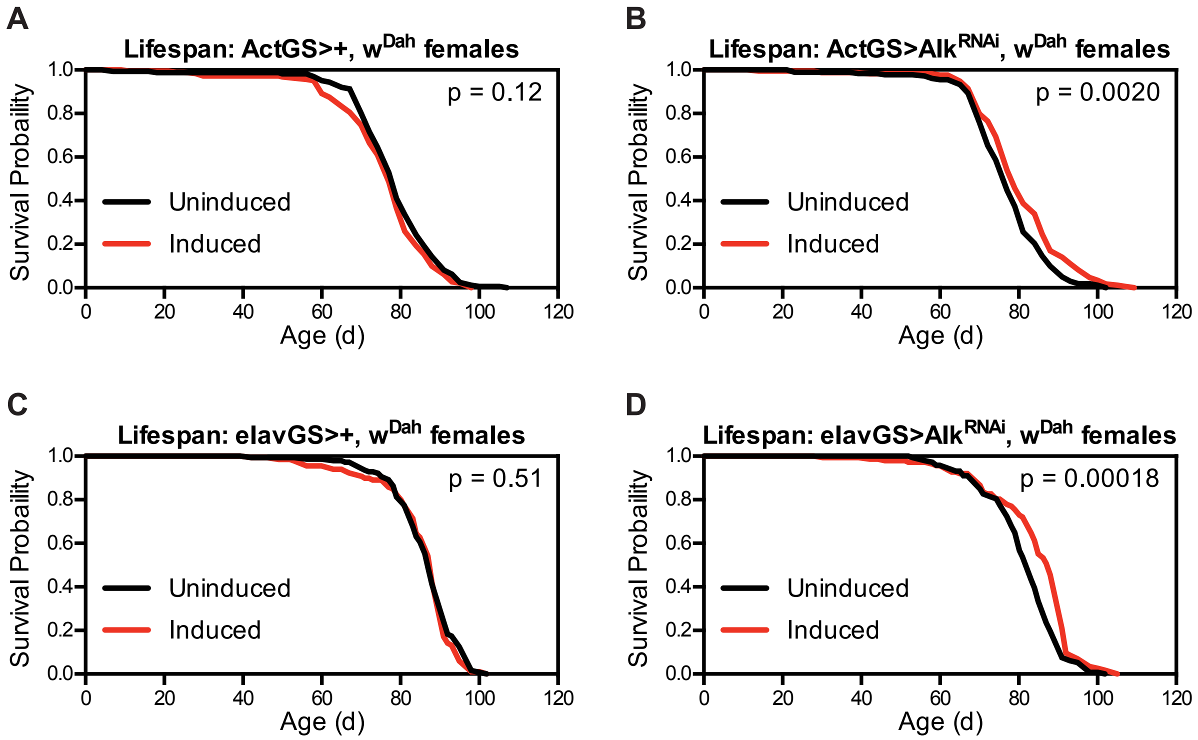

Supplementary Figure 2. RNAi knock-down of *Alk* ubiquitously or in neurons reproducibly extends lifespan. (**A**) Survival curves showed no significant change in lifespan for female w^{Dah};Act-GS/+ flies treated with RU-486 (200µM) from 2 days of age compared to sibling flies of the same genotype treated with vehicle control food. (**B**) Survival curves showed extended lifespan for female w^{Dah},UAS-Alk^{RNAi}/w^{Dah};Act-GS/+ flies treated with RU-486 (200µM) from 2 days of age compared to sibling flies of the same genotype treated with vehicle control food. (**C**) Survival curves showed no significant change in lifespan for female w^{Dah};;elav-$GS^{Tricoire}$/+ flies treated with RU-486 (200µM) from 2 days of age compared to sibling flies of the same genotype treated with vehicle control food. (**D**) Survival curves showed extended lifespan for female w^{Dah},UAS-Alk^{RNAi}/w^{Dah};;elav-$GS^{Tricoire}$/+ flies treated with RU-486 (200µM) from 2 days of age compared to sibling flies of the same genotype treated with vehicle control food. For all survival experiments, n>110 deaths counted per condition; p values are from log-rank tests versus the vehicle-treated (uninduced) group.

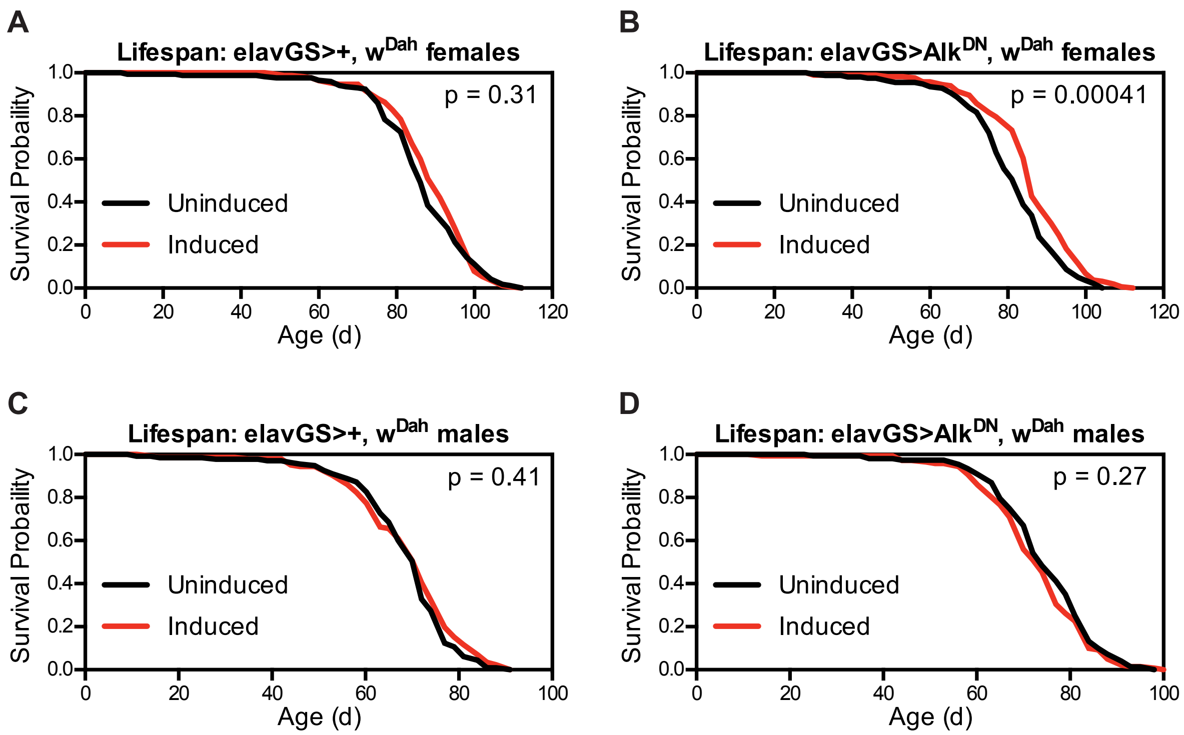

Supplementary Figure 3. Expression of dominant-negative *Alk* in neurons reproducibly extends lifespan in female but not male flies. (**A**) Survival curves showed no significant change in lifespan for female w^{Dah};;elav-GS^{301}/+ flies treated with RU-486 (200µM) from 2 days of age compared to sibling flies of the same genotype treated with vehicle control food. (**B**) Survival curves showed extended lifespan for female w^{Dah};;UAS-Alk^{DN}/elav-GS^{301} flies treated with RU-486 (200µM) from 2 days of age compared to sibling flies of the same genotype treated with vehicle control food. (**C**) Survival curves showed no significant change in lifespan for male w^{Dah};;elav-GS^{301}/+ flies treated with RU-486 (200µM) from 2 days of age compared to sibling flies of the same genotype treated with vehicle control food. (**D**) Survival curves showed no significant change in lifespan for male w^{Dah};;UAS-Alk^{DN}/elav-GS^{301} flies treated with RU-486 (200µM) from 2 days of age compared to sibling flies of the same genotype treated with vehicle control food. For all survival experiments, n>115 deaths counted per condition; p values are from log-rank tests versus the vehicle-treated (uninduced) group.

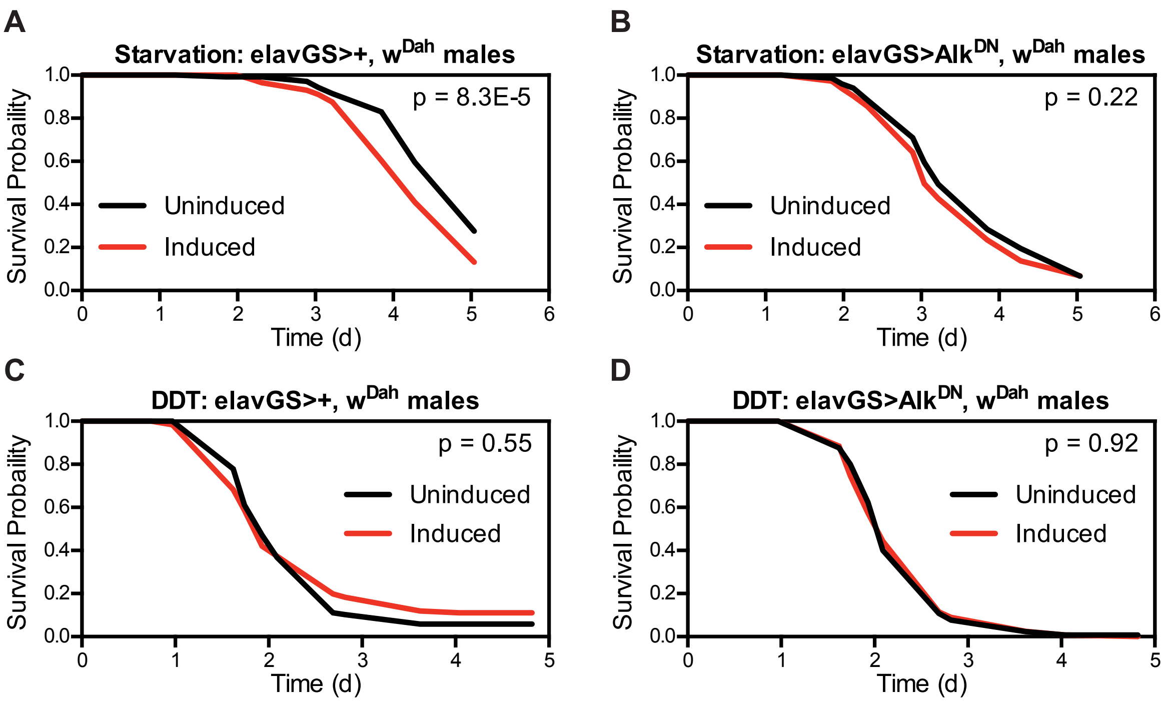

Supplementary Figure 4. Expression of dominant-negative *Alk* in neurons does not improve resistance to starvation or resistance to the xenobiotic toxin DDT in male flies. (**A-B**) Survival curves in starvation experiments started at 14 days of age showed (**A**) decreased survival for male w^{Dah};;elav-GS^{301}/+ flies and (**B**) no significant change in survival for male w^{Dah};;UAS-Alk^{DN}/elav-GS^{301} flies treated with RU-486 (200µM) from 2 days of age compared to sibling flies of the same genotype treated with vehicle control food. (**C-D**) Survival curves in DDT (0.03%) experiments started at 14 days of age showed (**C**) no significant change in survival for male w^{Dah};;elav-GS^{301}/+ flies and (**D**) no significant change in survival for male w^{Dah};;UAS-Alk^{DN}/elav-GS^{301} flies treated with RU-486 (200µM) from 2 days of age compared to sibling flies of the same genotype treated with vehicle control food. For all survival experiments, n>100 deaths counted per condition; p values are from log-rank tests versus the vehicle-treated (uninduced) group.

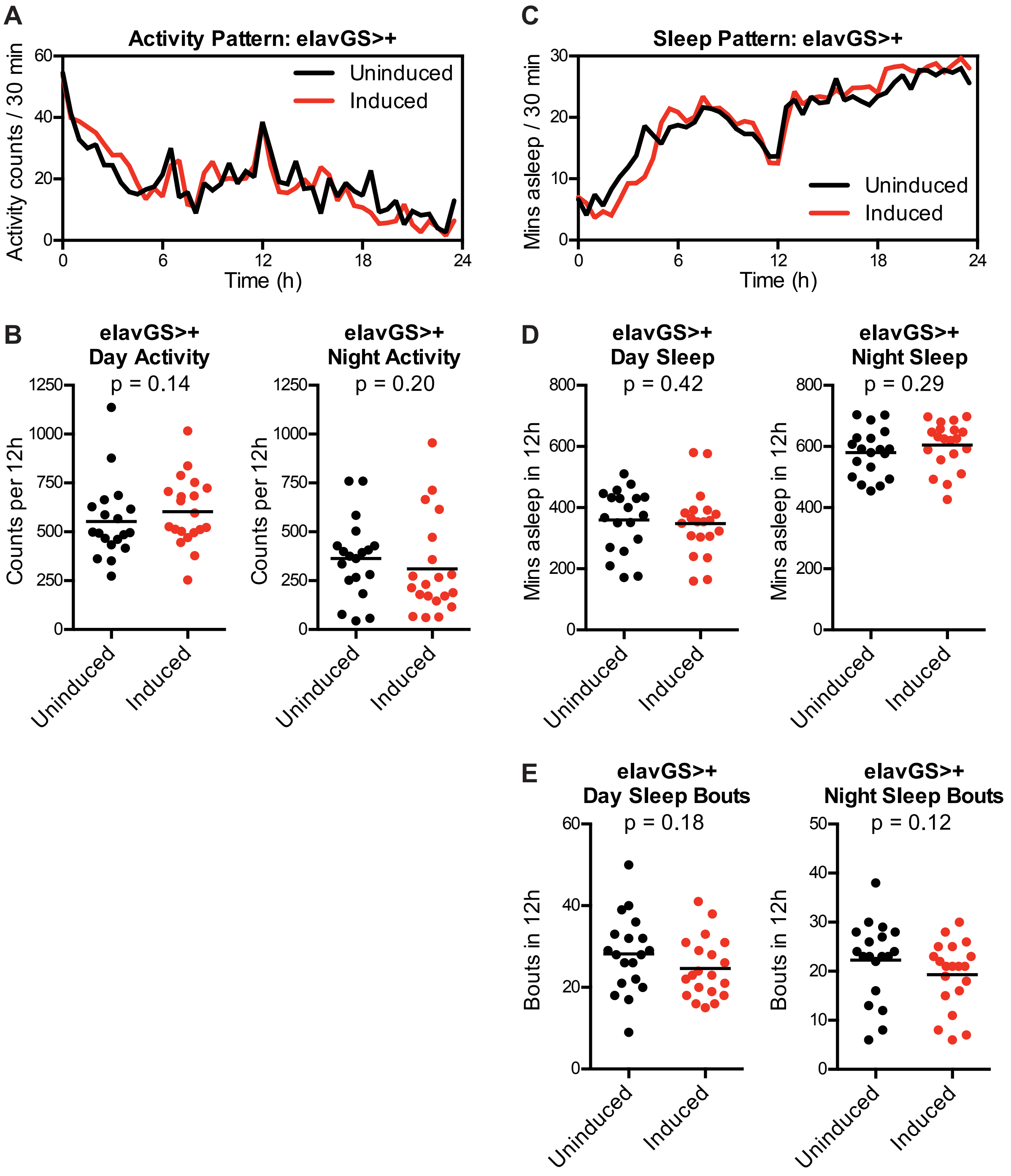

Supplementary Figure 5. Driver-alone elavGS/+ flies show no change in activity or sleep patterns with RU-486 treatment. (**A**) Activity traces and (**B**) quantification of day and night activity counts from mated female flies at 15 days of age showed no significant change in activity for w^{Dah};;elav-GS^{301}/+ flies treated with RU-486 (200µM) from 2 days of age compared to sibling flies of the same genotype treated with vehicle control food. (**C**) Sleep traces, (**D**) quantification of day and night sleep, and (**E**) quantification of the number of day and night sleep bouts from mated female flies at 15 days of age showed no significant change in activity for w^{Dah};;elav-GS^{301}/+ flies treated with RU-486 (200µM) from 2 days of age compared to sibling flies of the same genotype treated with vehicle control food. n=19-20 individual flies per condition; p values are from Mann-Whitney tests versus the vehicle-treated (uninduced) group.

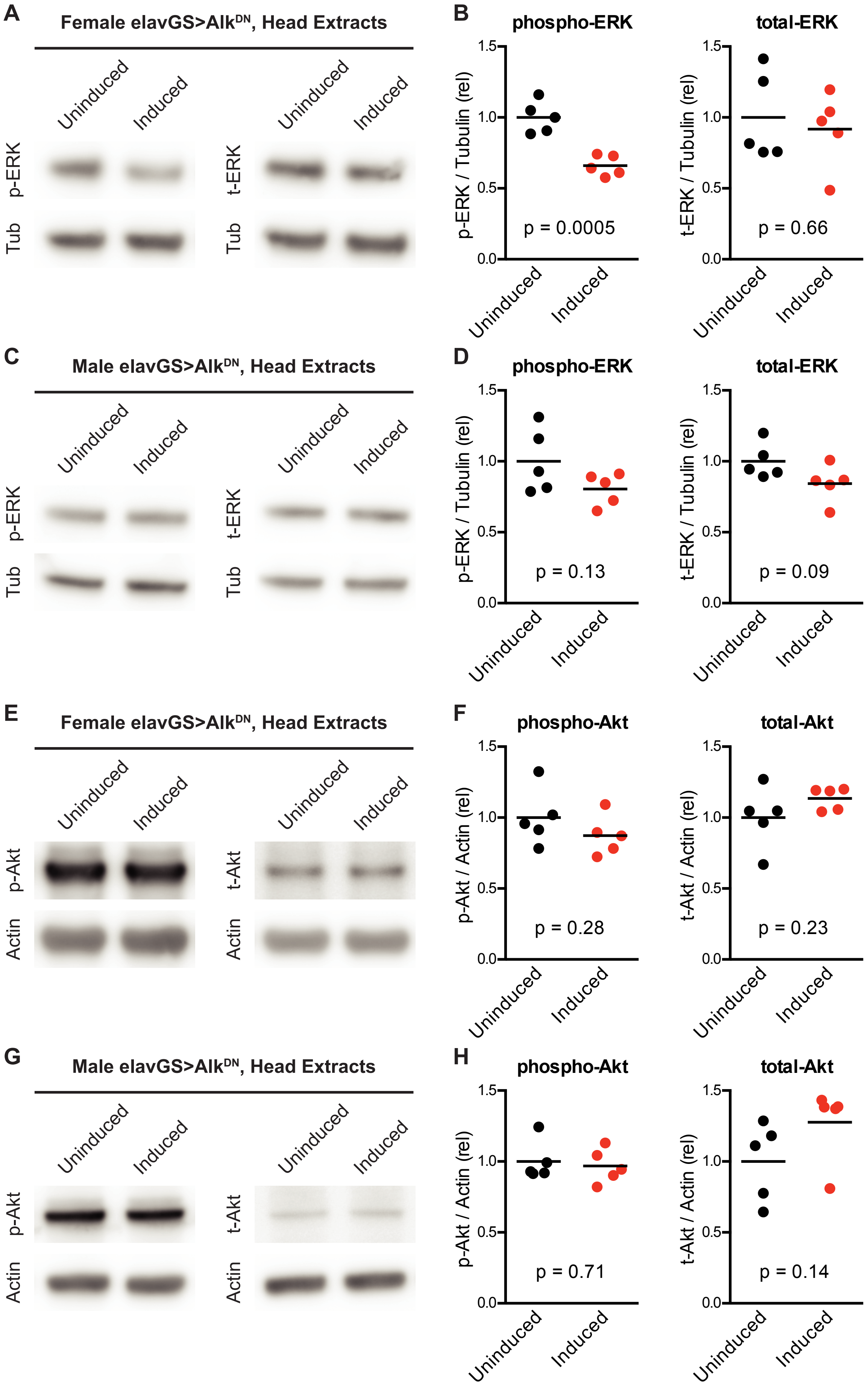
A
Female elavGS>Alk^DN, Head Extracts
Uninduced
Induced
p-ERK
Tub
t-ERK
B
phospho-ERK
total-ERK
p-ERK / Tubulin (rel)
t-ERK / Tubulin (rel)
p = 0.0005
p = 0.66
C
Male elavGS>Alk^DN, Head Extracts
D
p = 0.13
p = 0.09
E
Female elavGS>Alk^DN, Head Extracts
p-Akt
t-Akt
Actin
F
phospho-Akt
total-Akt
p-Akt / Actin (rel)
t-Akt / Actin (rel)
p = 0.28
p = 0.23
G
Male elavGS>Alk^DN, Head Extracts
H
p = 0.71
p = 0.14

Supplementary Figure 6 (previous page). Expression of dominant-negative *Alk* in neurons reduces Erk phosphorylation in females but not males, while not significantly affecting Akt phosphorylation. (**A-B**) Western blots from head extracts showed decreased levels of phosphorylated Erk and no significant change in the level of total Erk in female w^{Dah};;UAS-Alk^{DN}/elav-GS^{301} flies given food containing the inducing drug RU-486 (200μM) from 2 to 10 days of age compared to sibling flies of the same genotype treated with vehicle control food. (**C-D**) Western blots from head extracts showed no significant change in the levels of phosphorylated Erk or total Erk in male w^{Dah};;UAS-Alk^{DN}/elav-GS^{301} flies given food containing the inducing drug RU-486 (200μM) from 2 to 10 days of age compared to sibling flies of the same genotype treated with vehicle control food. (**E-F**) Western blots from head extracts showed no significant change in the levels of phosphorylated Akt (Thr342) or total Akt in female w^{Dah};;UAS-Alk^{DN}/elav-GS^{301} flies given food containing the inducing drug RU-486 (200μM) from 2 to 10 days of age compared to sibling flies of the same genotype treated with vehicle control food. (**G-H**) Western blots from head extracts showed no significant change in the levels of phosphorylated Akt (Thr342) or total Akt in male w^{Dah};;UAS-Alk^{DN}/elav-GS^{301} flies given food containing the inducing drug RU-486 (200μM) from 2 to 10 days of age compared to sibling flies of the same genotype treated with vehicle control food. n=5 biological replicates of 8 heads per replicate for each condition; p values are from unpaired t-tests between groups.

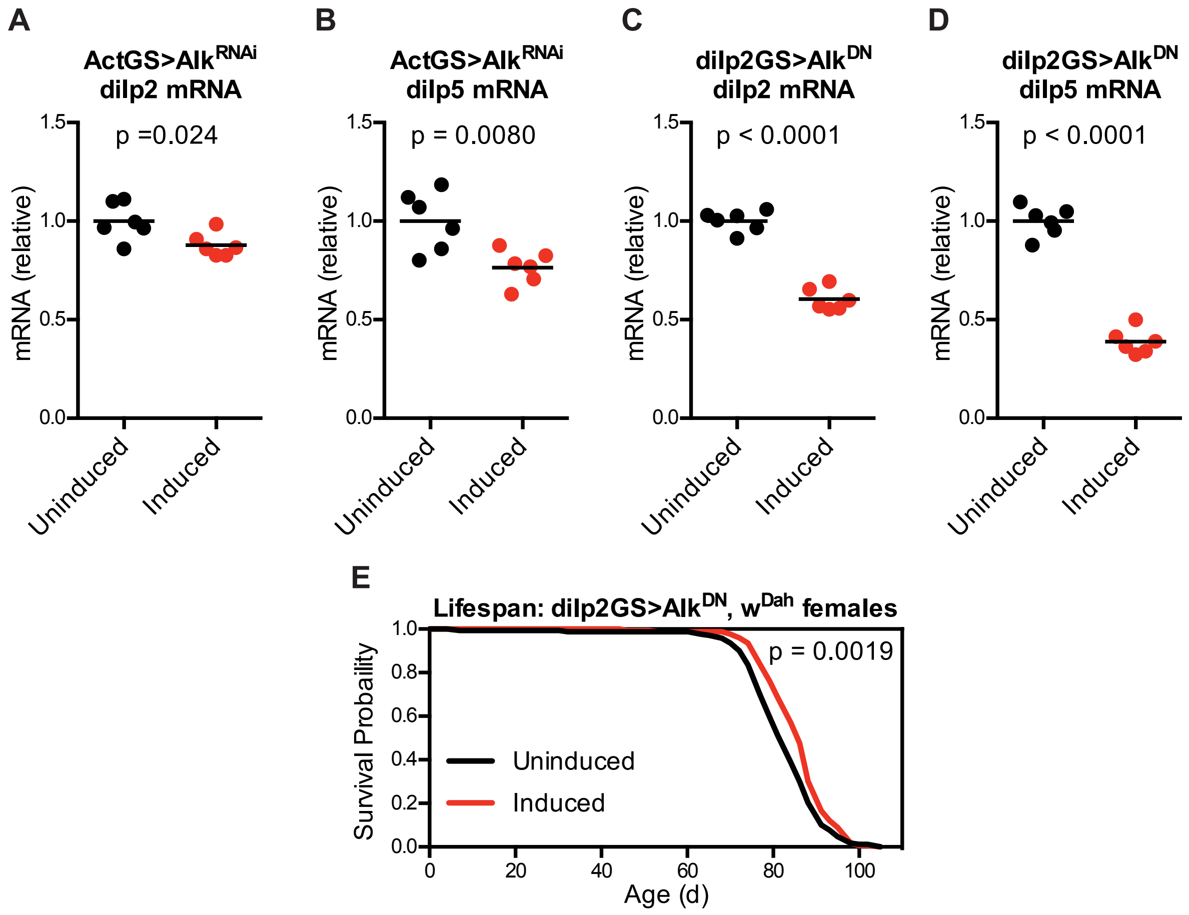

Supplementary Figure 7. RNAi knock-down of *Alk* or dilp-neuron-specific expression of dominant-negative *Alk* reduces mRNA levels for *dilp2* and *dilp5* and extends lifespan. (A-B) qPCR from head RNA showed decreased mRNA levels for *dilp2* and *dilp5* in female w^{Dah},UAS-Alk^{RNAi}/w^{Dah};Act-GS/+; flies given food containing the inducing drug RU-486 (200µM) from 2 to 11 days of age compared to sibling flies of the same genotype treated with vehicle control food. **(C-D)** qPCR from head RNA showed decreased mRNA levels for *dilp2* and *dilp5* in female w^{Dah};dilp2-GS/+;UAS-Alk^{DN}/+ flies given food containing the inducing drug RU-486 (200µM) from 2 to 11 days of age compared to sibling flies of the same genotype treated with vehicle control food. n=6 biological replicates of 6 heads per replicate for each condition; p values are from unpaired t-tests between groups. **(E)** Survival curves showed extended lifespan for female w^{Dah};dilp2-GS/+;UAS-Alk^{DN}/+ flies treated with RU-486 (200µM) from 2 days of age compared to sibling flies of the same genotype treated with vehicle control food. n>165 deaths counted per condition; p value is from log-rank tests versus the vehicle-treated (uninduced) group.

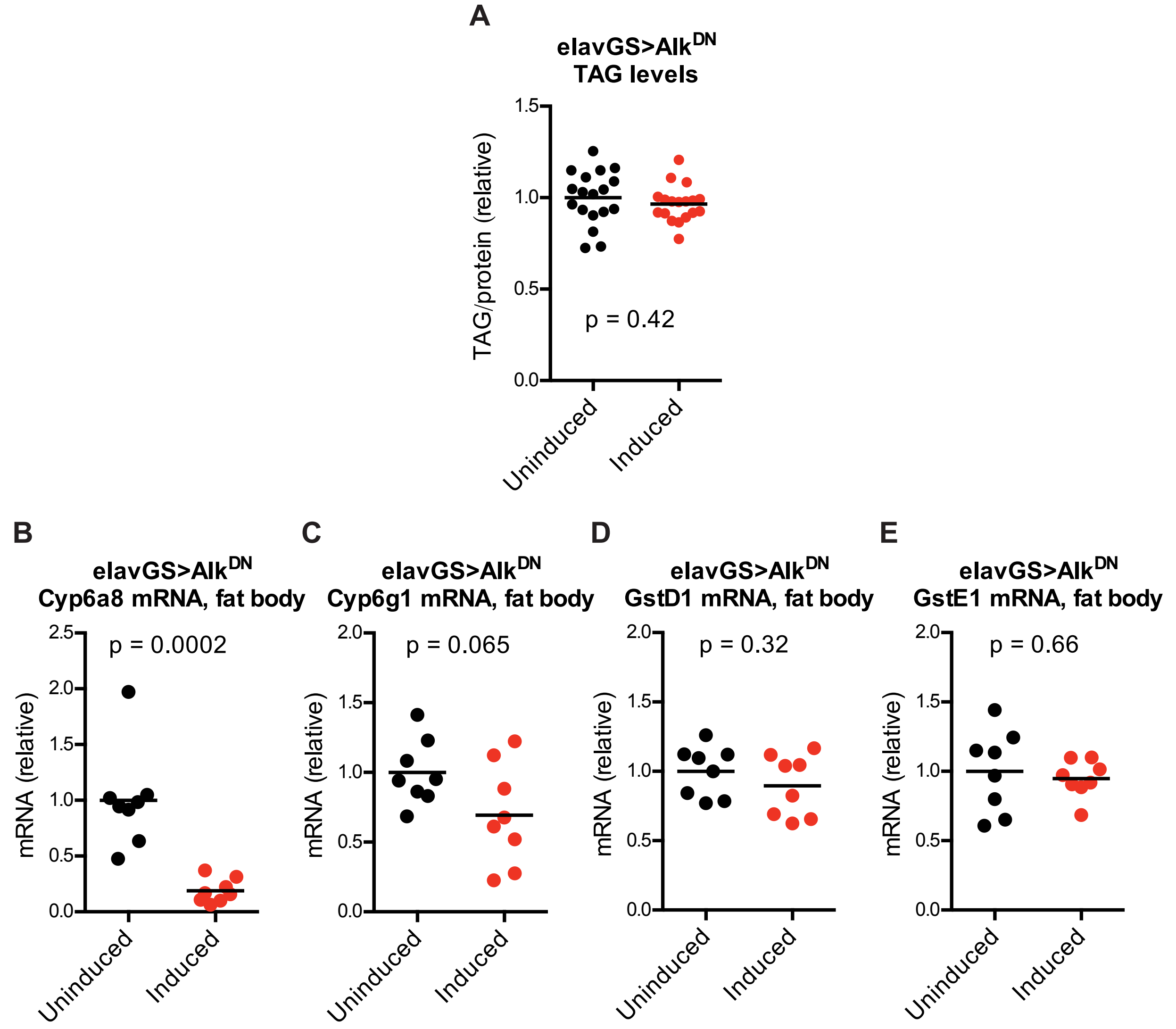

Supplementary Figure 8. Expression of dominant-negative *Alk* in neurons does not alter TAG levels but reduces mRNA levels for *Cyp6a8* in abdominal fat bodies. (**A**) Triglyceride (TAG) quantification from whole flies showed no change in TAG levels for female w^{Dah};;UAS-Alk^{DN}/elav-GS^{301} flies given food containing the inducing drug RU-486 (200µM) from 2 to 17 days of age compared to sibling flies of the same genotype treated with vehicle control food. n=18 individual flies for each condition; p values are from unpaired t-tests between groups. (**B-E**) qPCR from abdominal fat body RNA showed decreased mRNA levels for *Cyp6a8*, a trend towards decreased mRNA levels for *Cyp6g1*, and no change in mRNA levels for *GstD1* and *GstE1* in female w^{Dah};;UAS-Alk^{DN}/elav-GS^{301} flies given food containing the inducing drug RU-486 (200µM) from 2 to 18 days of age compared to sibling flies of the same genotype treated with vehicle control food. n=8 biological replicates of 4 abdominal carcasses per replicate for each condition; p values are from unpaired t-tests between groups.

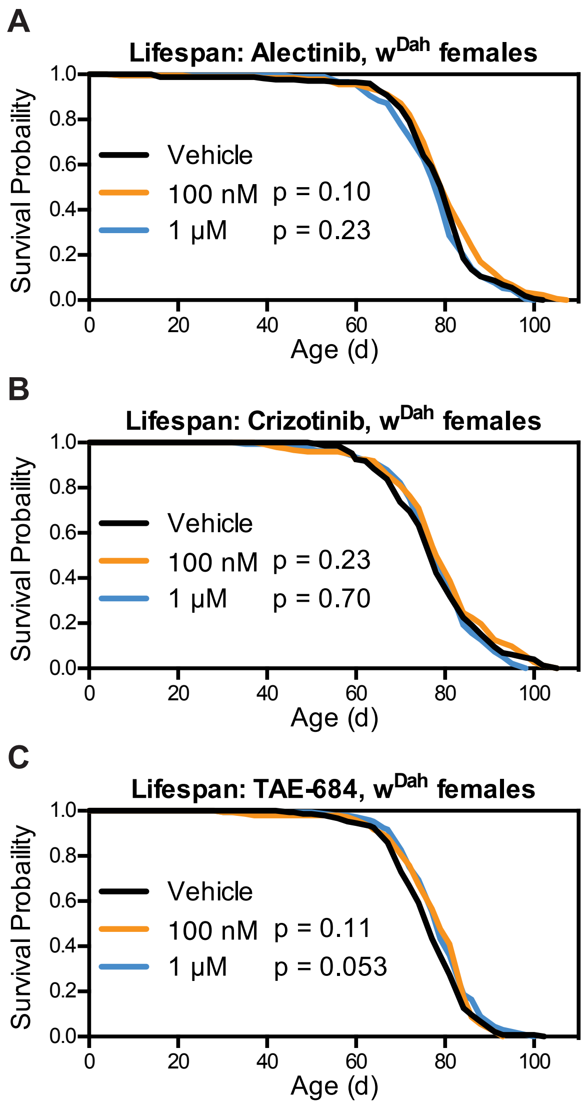

Supplementary Figure 9. The Alk inhibitors Alectinib, Crizotinib, and TAE-684 do not significantly extend lifespan at doses of 100nM or 1µM. Survival curves showed no significant change in lifespan for female w^{Dah} flies treated with (**A**) Alectinib (100nM or 1µM), (**B**) Crizotinib (100nM or 1µM), or (**C**) TAE-684 (100nM or 1µM) from 2 days of age compared to sibling flies of the same genotype treated with vehicle control food. Note that the vehicle control lifespan curves are the same as in Figure 7A-C, as these experiments were run in parallel. For all survival experiments, n>130 deaths counted per condition; p values are from log-rank tests versus the vehicle-treated group.

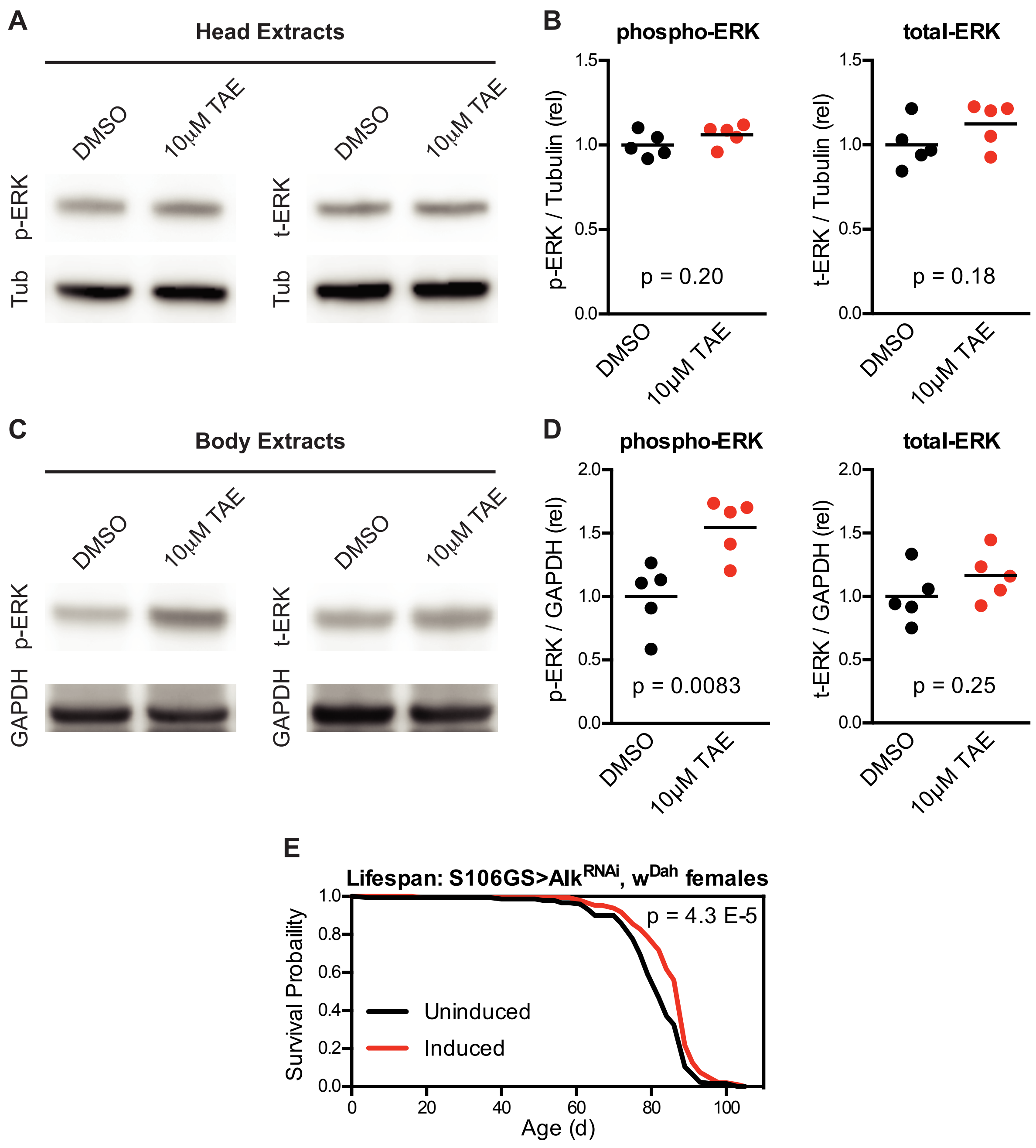

Supplementary Figure 10. Treatment with the Alk inhibitor TAE-684 alters Erk phosphorylation in body extracts, and RNAi knock-down of *Alk* in the fat body and gut extends lifespan. (**A-B**) Western blots from head extracts showed no significant change in the levels of phosphorylated Erk and total Erk in female w^{Dah} flies treated with TAE-684 (10μM) from 2 to 10 days of age compared to sibling flies of the same genotype treated with vehicle control food. (**C-D**) Western blots from headless body extracts showed increased levels of phosphorylated Erk and no significant change in the level of total Erk in female w^{Dah} flies treated with TAE-684 (10μM) from 2 to 10 days of age compared to sibling flies of the same genotype treated with vehicle control food. n=5 biological replicates for each condition; p values are from unpaired t-tests between groups. (**E**) Survival curves showed extended lifespan for female w^{Dah},UAS-Alk^{RNAi}/w^{Dah};$S_1$106-GS/+; flies treated with RU-486 (200μM) from 2 days of age compared to sibling flies of the same genotype treated with vehicle control food. n>130 deaths counted per condition; p value is from log-rank tests versus the vehicle-treated (uninduced) group.
